# Supplementary material for: GREEN: A lightweight architecture using learnable wavelets and Riemannian geometry for biomarker exploration with EEG signals
Source: Patterns (N Y). 2025 Feb 13;6(3):101182. doi: 10.1016/j.patter.2025.101182 (PMC11963017; doi:10.1016/j.patter.2025.101182)
Supplement: Document S1. Figures S1 and S2, Table S1, and Note S1 [file mmc1.pdf]

Patterns, Volume *6*

Supplemental information

GREEN: A lightweight architecture using learnable wavelets and Riemannian geometry for biomarker exploration with EEG signals

Joseph Paillard, Jörg F. Hipp, and Denis A. Engemann

Supplementary Materials - Paillard et al. – GREEN

Supplementary Note S1 - Manifold optimization

Given a SPD input matrix, an unconstrained `BiMap` layer will output a symmetric positive **semi**-definite matrix, which means it can have eigenvalues that are zero. To ensure that the output of the `BiMap` layer is a SPD matrix, with strictly positive eigenvalues, the weights of the `BiMap` can be constrained to be semi-orthogonal. To do so, we used the trivialization for gradient-based optimization on manifolds introduced by Lezcano-Casado[1]. This method consists in parametrizing a manifold in terms of a Euclidean space. In *Theorem 4.3*, the author shows that this approach is theoretically equivalent to using Riemannian gradients[2] which has been used in previous publications using Riemannian geometry and DL for EEG[3,4]. In addition to this theoretical result, we empirically observed that both approaches produce similar results. Given the improvement in computation time and reduced overhead, we opted for the trivialization approach.

Supplementary Results

Table S1: **Statistical comparison of G_2 and G_3 against** `baseline`**.** This table reports the detailed test statistics along with the median score, measured using balanced accuracy for all classification tasks and R^2-score for the age-regression.

**task**	**dataset**	**model**	**median score**	$\mathbf{t_{99}}$	`p-value`
age prediction	TUAB	G_3	0.7211	2.9906	0.0018
		G_2	0.6879	0.8650	0.1946
3-6 pathology classification	TUAB	G_3	0.8379	1.7064	0.0455
		G_2	0.8344	2.4251	0.0086
3-6 EO vs EC	TDBRAIN	G_3	0.8623	2.2305	0.01400
		G_2	0.8223	0.2184	0.4138
3-6 sex prediction	TDBRAIN	G_3	0.8325	-0.0491	0.4804
		G_2	0.8141	1.9805	0.0252
3-6 dementia diagnosis	CAU	G_3	0.6045	3.238332	0.0008
		G_2	0.5893	1.5949	0.0570

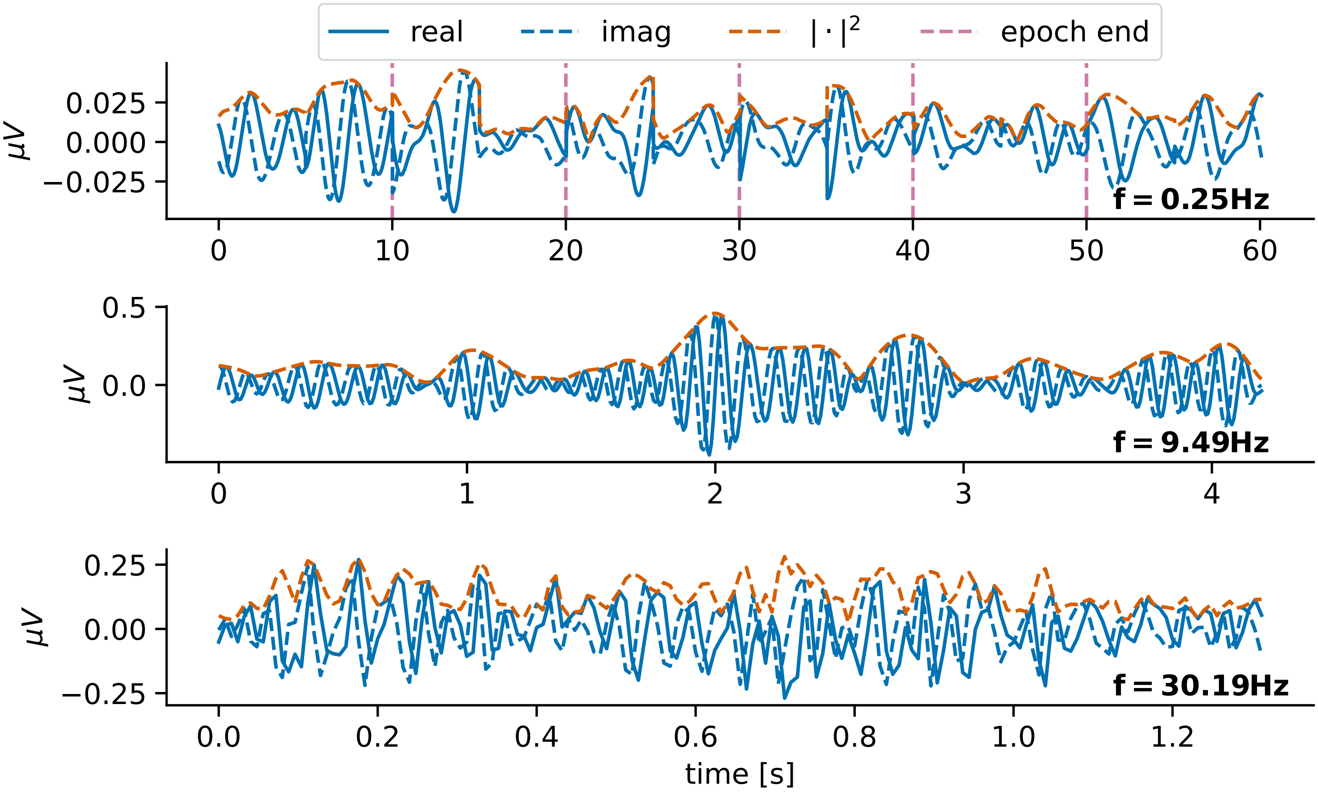

Figure S1: **Wavelet-transformed signal.** Each row represents the output of the convolution by one of the three complex-valued wavelets presented in Figure 5 (main text). The real and the imaginary parts are represented along with the squared modulus, sometimes referred to as power envelope. For the smallest frequency $(0.25Hz)$, multiple epochs have been represented side by side. This plot also reveals the small variations captured by the wavelet, despite the fact that the signal has been high-pass filtered above $1Hz$. This also suggests that task-relevant information is contained around this frequency, therefore pointing a sub-optimal preprocessing choice. The squared modulus variations are slower, evidencing its limited sensitivity to phase variations and small shifts.

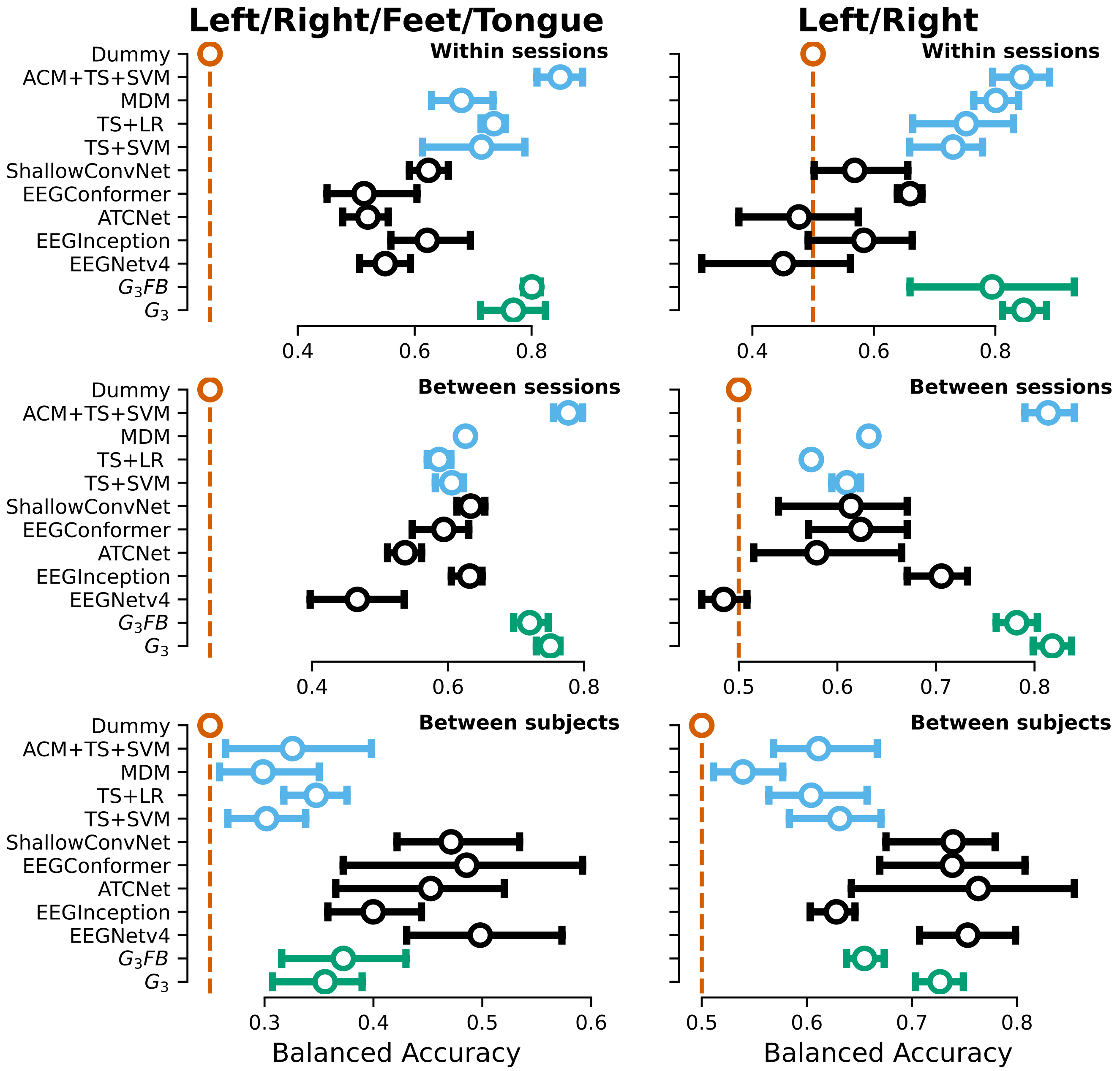

Figure S2: **BCI motor-imagery benchmarks by task and prediction mode (MOABB BNCI2014_001).** Riemannian and Deep architectures compared against GREEN models on motor imagery (left panels) and left/right movement (right panels) for prediction within sessions (first row), between sessions (middle row) and across subjects (bottom row). Chance-level prediction was estimated using a dummy model (red). GREEN models compared favorably for prediction within and between sessions, whereas more complex deep learning architectures showed dominance for motor imagery decoding across subjects. This advantage was less clear for left/right decoding across subjects.

References

1. Lezcano-Casado, M. (2019). Trivializations for Gradient-Based Optimization on Manifolds. arXiv. doi:10.48550/arxiv.1909.09501.

2. Absil, P.-A., Mahony, R., and Sepulchre, R. Optimization Algorithms on Matrix Manifolds. Princeton University Press (2008).

3. Carrara, I., Aristimunha, B., Corsi, M.-C., de Camargo, R. Y., Chevallier, S., and Papadopoulo, T. (2024). Geometric Neural Network based on Phase Space for BCI decoding. arXiv. doi:10.48550/arxiv.2403.05645.

4. Wilson, D., Schirrmeister, R. T., Gemein, L. A. W., and Ball, T. (2022). Deep Riemannian Networks for EEG Decoding. arXiv. doi:10.48550/arxiv.2212.10426.
