# Supplementary material for: GREEN: A lightweight architecture using learnable wavelets and Riemannian geometry for biomarker exploration with EEG signals
Source: Patterns (N Y). 2025 Feb 13;6(3):101182. doi: 10.1016/j.patter.2025.101182 (PMC11963017; doi:10.1016/j.patter.2025.101182)
Supplement: Document S2. Article plus supplemental information [file mmc2.pdf]

Article

Patterns

GREEN: A lightweight architecture using learnable wavelets and Riemannian geometry for biomarker exploration with EEG signals

Highlights

- GREEN combines deep learning (DL) with spectral analysis for EEG and geometric operations
- GREEN is compact and lightweight while performing like large DL models
- The modularity of GREEN allows integrating neuroscience domain knowledge
- GREEN facilitates learning interpretable EEG representations for biomarker exploration

Authors

Joseph Paillard, Jörg F. Hipp, Denis A. Engemann

Correspondence

joseph.paillard@roche.com (J.P.),
denis.engemann@roche.com (D.A.E.)

In brief

Our brains generate electrical signals whose unique patterns reveal information about aging, health, and even thoughts. This information is useful for studying brain disorders and developing novel therapies. However, traditional analysis methods can miss subtle but important brain-activity patterns. GREEN, introduced here, is an AI model that readily decodes brainwave patterns. By combining innovative mathematical tools with deep learning, GREEN detects subtle changes in brain activity with a low computational footprint while providing interpretable outputs that reveal insights into brain function.

Paillard et al., 2025, Patterns 6, 101182
March 14, 2025 © 2025 The Author(s). Published by Elsevier Inc.
https://doi.org/10.1016/j.patter.2025.101182

CellPress

Patterns

CellPress
OPEN ACCESS

Article

GREEN: A lightweight architecture using learnable wavelets and Riemannian geometry for biomarker exploration with EEG signals

Joseph Paillard,[1,2,*] Jörg F. Hipp,[1] and Denis A. Engemann[1,*]
[1]Roche Pharma Research and Early Development, Neuroscience and Rare Diseases, Roche Innovation Center Basel, F. Hoffmann–La Roche Ltd., Basel, Switzerland
[2]Lead contact
*Correspondence: joseph.paillard@roche.com (J.P.), denis.engemann@roche.com (D.A.E.)
https://doi.org/10.1016/j.patter.2025.101182

THE BIGGER PICTURE Electroencephalography (EEG) holds promise for developing brain-activity biomarkers advancing diagnosis, prognosis, and treatment of neurological and psychiatric disorders. Spectral analysis provides interpretable representations of the EEG by decomposing the signals into underlying frequency-specific oscillatory activity. This enabled established clinical applications of EEG (e.g., monitoring epileptic seizures, depth of anesthesia, or defining sleep stages). These methods may fall short in capturing more subtle EEG patterns, which may be the key to novel applications of EEG. Across scientific disciplines, including neuroscience, deep learning (DL) has emerged as a versatile framework for uncovering hidden patterns from complex high-dimensional data. There remains a critical gap in terms of combining the predictive capacity of state-of-the-art DL models with the interpretability of established EEG signal-processing methods, which have proven useful for expressing neuroscientific knowledge.

SUMMARY

Spectral analysis using wavelets is widely used for identifying biomarkers in EEG signals. Recently, Riemannian geometry has provided an effective mathematical framework for predicting biomedical outcomes from multichannel electroencephalography (EEG) recordings while showing concord with neuroscientific domain knowledge. However, these methods rely on handcrafted rules and sequential optimization. In contrast, deep learning (DL) offers end-to-end trainable models achieving state-of-the-art performance on various prediction tasks but lacks interpretability and interoperability with established neuroscience concepts. We introduce Gabor Riemann EEGNet (GREEN), a lightweight neural network that integrates wavelet transforms and Riemannian geometry for processing raw EEG data. Benchmarking on six prediction tasks across four datasets with over 5,000 participants, GREEN outperformed non-deep state-of-the-art models and performed favorably against large DL models while using orders-of-magnitude fewer parameters. Computational experiments showed that GREEN facilitates learning sparse representations without compromising performance. By integrating domain knowledge, GREEN combines a desirable complexity-performance trade-off with interpretable representations.

INTRODUCTION

Electroencephalography (EEG) is well established for assessing large-scale cortical dynamics[1] and has proved useful for analysis of sleep,[2] anesthetic monitoring,[3] and clinical examination of seizures.[4] These applications have in common that the phenomena of interest are characterized by high-amplitude signals that can be visually assessed by trained clinicians. Over the past decades, research in clinical and cognitive neurosciences has led to a wide range of experimental methods and data-analysis strategies that have allowed researchers to quantify patterns of brain activity that are hard to detect from raw EEG traces.[5–11] Furthermore, a coevolution of instrumentation,[12,13] curation of reusable datasets,[14–16] and innovation in signal processing and machine learning (ML)[17] unfolded within the field of EEG. In combination, these factors hold promise to extend the clinical and biotechnological applications of EEG by facilitating the discovery of novel brain-activity biomarkers.

In many traditional EEG applications, the signal power at specific electrodes or the global average signal power have often

© 2025 The Author(s). Published by Elsevier Inc.
This is an open access article under the CC BY license (http://creativecommons.org/licenses/by/4.0/).

been sufficient for monitoring and phenotyping patient groups based on changes in oscillatory brain activity.[18–20] The logarithm of the power is often preferred as it reflects universal scaling laws of brain structure and function.[1,21] The logarithm also facilitates comparisons between frequencies along the spectrum, which, due to inverse proportionality between power and frequency (the $1/f$ scaling), is dominated by slower brain waves.[22,23] When the objective is to build predictive models of complex cognitive processes, pathologies, or other biomedical variables, modeling approaches that make more elaborate use of the multivariate complex EEG signal may substantially enhance the signal-to-noise ratio.[24,25] Examples include motor imagery in brain-computer interfaces (BCIs),[26,27] subtle signal alterations related to disorders of the central nervous system (CNS),[28] or pharmaceutical treatments.[29,30]

A fundamental problem that needs to be faced in such applications arises from electromagnetic field spread. Due to volume conduction, electrical potentials are spread out across all electrodes such that signals measured on the scalp—in the electrode space—imply non-linear distortions[31–34]; e.g., electrode-space power distributions do not stand in a linear relationship to the source-power distributions of interest. Source localization is a method for uncovering local and long-range neural synchronization by unmixing the EEG signal by solving biophysical inverse problems before computing features.[9,35–37] However, this is not always practical as source localization is most accurate with individual anatomic magnetic resonance imaging (MRI) and additional interactive work for coregistration between electrodes and the individual brain model. Furthermore, in a distributed brain model, thousands of candidate dipole locations are used, which can add complexity and often requires the choice of anatomical atlases for dimensionality reduction.[38–40]

In this context, ML has provided powerful tools for uncovering complex brain signals arising from multichannel EEG recordings. For instance, by learning spatial filters using the between-channel covariance matrix as input, the problem of volume conduction and source mixing can be tackled without explicitly relying on biophysical source models.[30,33,41] The same approach can be used for enhancing local oscillatory patterns and subtracting background noise.[24] Alternatively, Riemannian geometry provides a theoretical framework to derive representations for covariance matrices, which mitigate distortions due to volume conduction by projecting them to a Euclidean space while preserving distances, hence allowing the use of classical ML tools.[42,43] When preceded by frequency filtering, these features can be used with standard regularized linear techniques to define models for predicting from subtle brain-activity patterns.[34]

More recently, the rich literature of deep learning (DL) for speech processing and computer vision has been repurposed for the analysis of EEG signals, leading to several potential benefits.[44–46] First, DL models allow for end-to-end computation (i.e., combining temporal filtering, spatial filtering, and estimation of non-linear functions into one optimization problem).[47,48] Furthermore, optimizing the parameters of these models via stochastic optimization methods[49,50] makes it possible to learn from large datasets despite the significant memory footprint of the raw signal. Finally, the huge functional space covered by these architectures combined with their modular nature enables learning complex functions, which holds promise to facilitate the discovery of previously neglected signals.

Despite major methodological advances, DL applied to EEG remains to unlock breakthroughs in clinical applications or biomarker discovery. In the EEG context, we have identified several limitations of the applied DL literature that can hinder the attractiveness of DL methods for neuroscientists. First, the architecture choices are most of the time directly adopted from speech processing or computer vision applications.[15,51,52] As a consequence, the complexity of models increased along with data requirements and computational cost while missing an intelligible link with established neuroscientific results and concepts such as capturing the oscillatory dynamics of neural populations in the cortex using band-pass filtering or measuring phase coupling.[1,9,53] This makes it more difficult to understand how the functions learned by DL relate to the EEG landmarks and phenomena investigated in the neuroscience literature, hence limiting the utility as a tool in applications.

Parallel research efforts have led to first successes at incorporating established EEG processing operations. For example, while still applying a canonical convolutional architecture design, Schirrmeister et al. proposed a minimal architecture that mimicked the operations of classical common-spatial-pattern filter-bank models.[41] In a recent study, real-valued wavelets were used instead of classical convolution kernels.[54] Another line of research has studied Riemannian geometry in DL models.[55–58] It is, however, noteworthy that several DL architectures explored for EEG focus on the power of the signal.[47,54,55] Such DL models do not explicitly compute phase-related features; hence, they potentially miss the connection with the vast EEG literature on phase coupling.[59,60] Finally, the lack of constraints in these architectures can provoke unsustainable computational scaling requiring massive datasets that are not yet available for EEG.[61,62] To support further exploration of the untapped potential of EEG for medical applications and biomarker development through DL, it will, therefore, be important to integrate neuroscientific and biophysical prior knowledge into the architecture design of neural networks.

In this work, we make an attempt to address this challenge. We propose a new lightweight architecture termed Gabor Riemann EEGNet (GREEN), combining Gabor wavelets and Riemannian computation in a neural network for EEG data. The article is organized as follows. We first present a background review of the related literature, motivate the architecture choices, and detail the different architectures studied in this work. We then present empirical benchmarks of model performance and complexity on three international datasets with EEGs from more than 5,000 human participants in five prediction tasks. These benchmarks focus on studying improvement over robust and state-of-the-art baseline models based on Riemannian geometry as well as positioning our work against DL architectures. Finally, we demonstrate the utility of GREEN for exploring model complexity in terms filter-bank sizes and learned representations in different prediction tasks.

RESULTS

In this work, we developed a novel architecture for the end-to-end processing of raw EEG data combining the extraction of

CellPress
OPEN ACCESS

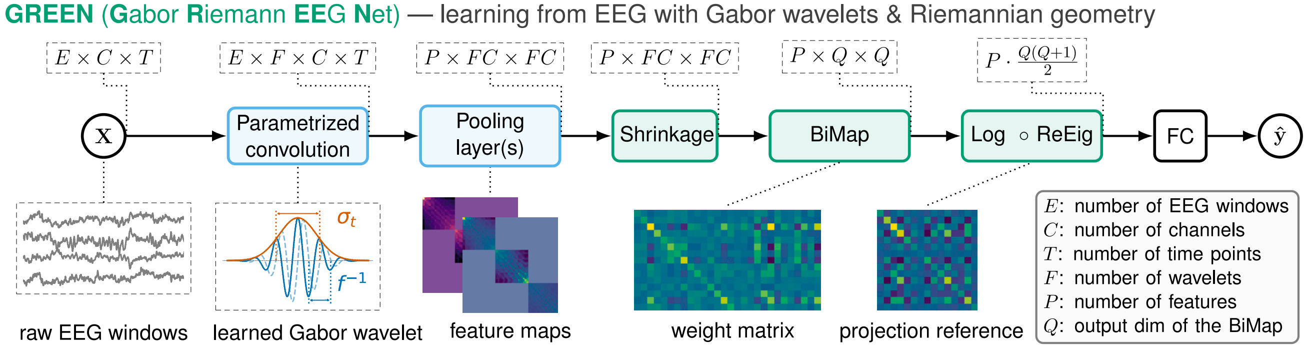

Figure 1. Composable GREEN building blocks and model architecture

The input consists of randomly drawn windows from EEG recordings (E windows, each with T time points). Blocks colored in blue operate on inputs that have a temporal dimension, whereas blocks colored in green operate on matrices. In the first convolution block, the kernel is a parametrized complex-valued Gabor wavelet. The parameters of this convolution layer are the standard deviation σ_t of the Gaussian window and the central frequency f of the complex sinusoid. The pooling layer comprises the computation of the covariance matrix from the wavelet-transformed signal. Using equal wavelet lengths ensures the consistency of wavelet-transformed signals and allows us to evaluate cross-frequency interactions between channels. The pooling layer can accommodate other features derived from wavelet-transformed signals (e.g., pairwise phase-locking value, the number of features being denoted P). The following layer performs a shrinkage operation on the symmetric positive semi-definite feature matrices, leading to down-weighing off-diagonal adapting a Ledoit-Wolf scheme[63] where the shrinkage parameter is trainable. The subsequent bilinear mapping (BiMap) and rectified eigenvalue (ReEig) layers have been introduced elsewhere[64] and provide computations on the Riemannian manifold, including spatial filtering, dimensionality reduction, and eigenvalue non-linearity. We modified the original implementation of the LogEig layer. Instead of the identity, a log-Euclidean running mean is used as reference point for the projection. This provides a meaningful reference point accounting for the dominance of spatial patterns induced by electromagnetic field spread and volume conduction. Finally, the tangent-space vectors are fed to fully connected (FC) layers that can be designed and adapted for specific modeling goals. We benchmarked variations of the GREEN architecture based on a subset of these building blocks (Table 1), which allowed us systematically exploring hypotheses on data-generating mechanisms.

time-frequency features with learned filter banks of Gabor wavelets, geometric transformations, and fully connected layers (Figure 1). For details on architecture and a review of related prior work, see section Methods. For architecture variations benchmarked in this work, see Table 1.

The impact of architecture components depends on the prediction task

We first explored different architecture components and evaluated their performance on five tasks and three datasets with available benchmarks (Figure 2). The five benchmarks include age regression on the Temple University Hospital Abnormal (TUAB) dataset, binary classification of pathological versus normal recordings using the TUAB dataset, binary sex classification on Two Decades Brainclinics Research Archive for Insights in Neurophysiology (TDBRAIN) dataset, binary classification of recordings with eyes open versus eyes closed from the TDBRAIN dataset, and three-way classification of diagnosis (healthy, mild cognitive impairment [MCI], or dementia) using the Chung-Ang University (CAU) dataset. By design, the modularity of the GREEN architecture supported this objective by allowing us to remove or freeze the parameters in order to reproduce the operations performed by baseline methods and assess the benefits of additional complexity (cf. Figure 1 and Table 1).

We first revisited age prediction on the TUAB (Figure 2A), which has been intensely studied with Riemannian filter-bank regression in prior work.[34,66,67] Results are shown for three different models. G_1 reproduces the operations of the baseline, including a pooling layer that computes only the covariance ($P = 1$) and a BiMap layer but uses a stochastic optimization method. The input dimension of the BiMap depended on the number of channels C (see section Datasets and preprocessing) and the output dimension was set to $Q = C - 1$. G_2 has an additional hidden layer (32 units). G_3 uses a learnable filter bank of $F = 10$ wavelets and two BiMaps of respective output dimensions $Q = 64$ and $Q = 32$. For more details, refer to Table 1. Our results showed that the addition of a hidden layer improved performance by a few percent (not statistically significant). This comparison is meaningful to empirically test the logarithm hypothesis motivated by insights into log-dynamic scaling of brain structure and function.[21,34] For the case of age prediction, results suggests that the logarithm is probably a sufficient modeling assumption to solve the age-prediction task. Importantly, the greater improvement was observed for model G_3, which introduced filter banks of trainable wavelets, suggesting that task-relevant information can be better captured with learned filters rather than a fixed grid.

We extended the comparison between the baseline, G_2, and G_3 architecture to the four remaining classification tasks (Figure 2B). The results showed that the relative improvement over the baseline varied depending on the task, confirming the intuition that the spread of information along the frequency spectrum may be task dependent. Improvements with G_2 were significant ($\alpha = 0.05$) for sex prediction and pathology decoding. Improvements with G_3 were significant ($\alpha = 0.05$) for eyes-closed versus eyes-open classification, pathology decoding, and dementia classification. For a detailed report of performance scores and test statistics, see Table S1.

The benefits of learnable wavelets was most pronounced for dementia and eyes-closed versus eyes-open classification as indicated by cross-validation uncertainty and average performance differences. Furthermore, a predefined filter bank can lead to similar (pathology decoding) or even better (sex prediction) performance than a learned filter bank and, likewise, the

Table 1. Architecture details for the different models studied

Input	Raw		Feature map		Vector		–
Component	Filter Bank	Pairwise PLV	BiMap	LogMap	Linear	Hidden layer	Adam
Model	–	–	–	–	–	–	–
Baseline	✗	✗	✗	✔	✔	✗	✗
G_1	✗	✗	✔	✔	✔	✗	✔
G_1 P	✗	✔	✔	✔	✔	✗	✔
G_2	✗	✔	✗	✔	✔	✔	✔
G_3	✔	✔	✔	✔	✔	✔	✔
G_3 P	✔	✔	✔	✔	✔	✔	✔

more complex fully connected (FC) layers can outperform the logarithm non-linearity (sex prediction). These results emphasize the importance of a modular approach to build models that can be adapted to the task at hand.

GREEN shows a favorable performance-complexity trade-off while remaining extensible

The CAU dataset[15] offered us the opportunity to position the proposed models on a benchmark for diagnosis prediction in dementia against recent large DL architectures (Figure 3A). Replotting the results from Kim and colleagues[15] revealed a log-linear scaling of performance with model size, in number of parameters (Pearson correlation coefficient of 0.98). In contrast, proposed models based on Riemannian geometry (Rn, model G_1, G_1P) seemed to break this trend and presented a favorable performance-complexity trade-off. We observed that increasing the complexity of the model by allowing the learning of the parameters of 10 wavelets (Rn + Wav, models G_3 and G_3P), the performance was largely improved, outperforming the best single model presented in the original paper and matching the performance of the ensemble model (bag of all the other models presented in the paper) (Figure 3A). These performance gains were achieved with up to three-orders-of-magnitude fewer parameters than other DL models.

Availability of prior information that the recordings contained segments of photic stimulation, which has the capacity to alter neural phase synchrony in stimulation,[69–71] motivated us to add a widely used measure of phase synchrony,i.e., pairwise phase-locking value (PLV)[59] matrices, in the pooling layer (see Equation 4). The lean design of the proposed GREEN architectures allowed us to investigate the hypothesis regarding the relevance of phase-locking features more rigorously by running model comparisons with a full-blown Monte-Carlo cross-validation scheme with 100 repetitions (unfortunately not available for the original CAU benchmark). When concatenating the covariance matrices with PLV matrices, the performance was improved significantly for G_1P over G_1 and slightly for G_3P over G_3 (Figure 3B), suggesting that phase-locking measures were informative but not indispensable for the more complex architecture with trainable wavelets.

We next investigated scaling performance and computation time using learning curves compared against the deep EEGConformer architecture.[68] For model performance, we observed highly similar scaling across increasing fractions of training data used (Figure 3C). On the other hand, for computation time, non-linear scaling differences emerged between GREEN and the EEGConformer (Figure 3D). We explored these trends using linear regression analysis modeling log computation time as an additive function train fraction, model, and their interaction. We found a significant effect of fraction ($\beta = 0.0099, SE = 0.001$, $t(8) = 13.451, p < 8.945 \times 10^{-7}$) and a significant interaction effect between fraction and model with GREEN as reference model ($\beta = 0.0059, SE = 0.001, t(8) = -5.6, p < 0.0005$). Considering log time, this means that, for the EEGConformer, computation time increased by $10^{0.0099} = 2.3\%$ for 1% increase in training-data fraction. On the other hand, for GREEN, this increase was by $10^{-0.0059} = -1.3\%$ slower, leading to more than 400% advantage as all data points were used.

These experiments demonstrate two key features of the GREEN architecture. First, it improves the performance-complexity trade-off by integrating prior knowledge in the architecture, through parameterized wavelets and Riemannian computations, which alleviates the size of models without reducing the prediction accuracy. This allows it to outperform large DL models with orders-of-magnitude fewer parameters. Second, the modularity of the framework facilitates the integration of classical neuroscience measures of phase synchrony. This facilitates testing of specific scientific hypotheses regarding the predictive importance of, e.g., phase synchrony features in the context of EEG recordings containing photic stimulation periods.

The optimal numbers and frequencies of wavelets depends on the prediction task

Our results revealed how the proposed architecture can lead to performance benefits at a low computation cost. In this context, the size of the filter bank is an intuitive hyperparameter, measuring the spread of the information along the frequency spectrum. This raises the question to what extent task-relevant information is concentrated versus distributed along the frequency spectrum. The filter bank that presents the optimal trade-off between size and performance could thus be seen as a measure of the model complexity needed to solve a task.

We investigated the minimal filter bank size needed to reach the performance of the most complex model. For each task, we progressively increased the filter-bank size using $F = 1, \cdots, 15$ wavelets and tracked the performance on the same single testing fold; error bars (SD) are measured for five random initializations (Figure 4A). For each task, the curve was plotted until the model achieved a performance less than one standard deviation away from the model with the largest filter bank ($F = 15$), which consistently yielded the best results. It can be seen that the number of wavelets needed to reach this

CellPress
OPEN ACCESS

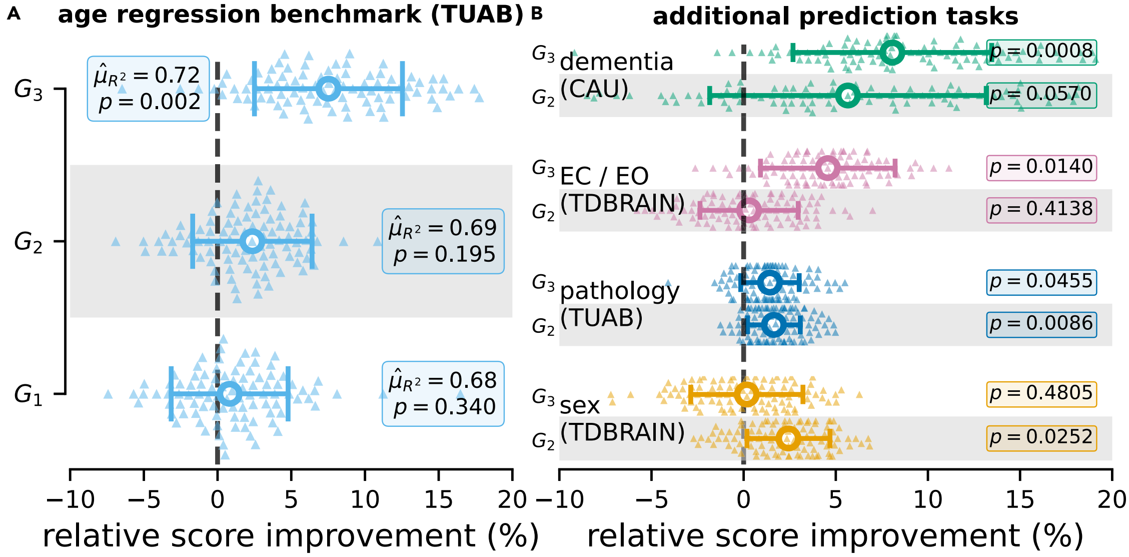

Figure 2. The impact of GREEN architecture components varied across prediction tasks

(A) Cross-validated performance (100 Monte-Carlo splits, 20% test set size) for age regression on TUAB data (healthy subjects) for models of increasing complexity ($G_1 - 3$, cf. Table S1). Error plots (SD) show distributions of percentage changes of R^2 between each model versus the baseline. p values obtained form Nadeau's and Bengio's corrected t test.[65] Annotations show the absolute score of the model ($G_1 - 3$) and hypothesis tests (null hypothesis being no improvement over baseline). G_1 and G_2 used the same filter bank as the baseline. G_1 mirrors the non-deep baseline but uses a stochastic optimization procedure. G_2 added a hidden layer enabling non-linear functions beyond the logarithm implied by the tangent-space embedding. In addition to the hidden layer, G_3 also used trainable wavelets and takes raw EEG as input. G_3 showed markedly higher performance than G_1, suggesting that fine-tuning of frequency filtering and non-linearities were beneficial for this task.

(B) Same experimental protocol and plotting convention as in (A) for four additional tasks (from top to bottom): three-way classification of diagnosis (healthy, mild cognitive impairment [MCI], or dementia), binary classification of recordings with eyes open (EO) versus eyes closed (EC), of pathological versus normal recordings, and of the patient's sex. For each task, the dataset used is specified in parentheses. The advantage of the architectures G_3 depended on the task and was most pronounced for dementia diagnosis and classification of EO versus EC.

best performance was highly task dependent, providing insights into the model complexity requirements.

The results for the dementia diagnosis and the eyes state tasks revealed that the minimal filter-bank size was only two or three wavelets. On the other hand, for age- and sex prediction, more wavelets were required, which, in the case of sex prediction, did not transform into improved performance over the baseline. This suggests that the predictive spectral signatures on certain prediction tasks were more localized and less diffuse in the frequency spectrum. In these cases, the G_3 learned to extract sparse representations of the complex EEG signals that concentrated the information needed to solve prediction tasks.

We then inspected the center frequencies of the wavelets learned by the model with a sparse filter bank for each of the five tasks studied (Figure 4B). Such frequencies could be interpreted as the most predictive and least redundant spectral features of the signal. Across tasks, the low-frequency region of the spectrum was often selected alongside the alpha band (8–12 Hz), which is a strong signature of wakeful human EEG and has been intensely studied due to its favorable signal-to-noise ratio.

These results highlight a strength of the proposed architecture to uncover the informative frequency ranges via supervised learning. To the contrary, classical pipelines rely on predefined filter banks and require a far more fine-grained coverage of the frequency spectrum to solve certain tasks, leading to larger filter banks. In sum, such a profiling benchmarks can provide important leads for in-depth exploration of underlying predictive EEG signatures and task-specific markers.

GREEN yields interpretable and actionable EEG quantities for biomarker exploration

The modular design of GREEN, which is based on scientifically interpretable components, encourages further in-depth exploration and extraction of reusable EEG quantities (Figure 5). As wavelets can be interpreted as band-pass filters, it can be seen that the diagonal of the covariance matrix contains classical EEG-power topographies. To investigate the utility of GREEN for data exploration, we investigated the eyes-closed versus eyes-open prediction task in more detail. Since Hans Berger's discovery of the EEG, it has been known that closing the eyes predictably intensifies occipital alpha rhythms that have been linked across species to sensory idling functions and "partial disengagement from the environment."[72] This well-known phenomenon is thus a good toy example to study the utility of the GREEN architecture to probe physiological understanding. It can be seen that G_3 places one wavelet exactly in the expected 8 to 12 Hz region, where the power difference is most visible (Figure 5A). In addition, model selection showed that three wavelets were needed to achieve high performance. It is noteworthy that the frequency-domain standard deviation is minimal for the wavelet located in the alpha band. This is a substantial deviation from the popular Morlet

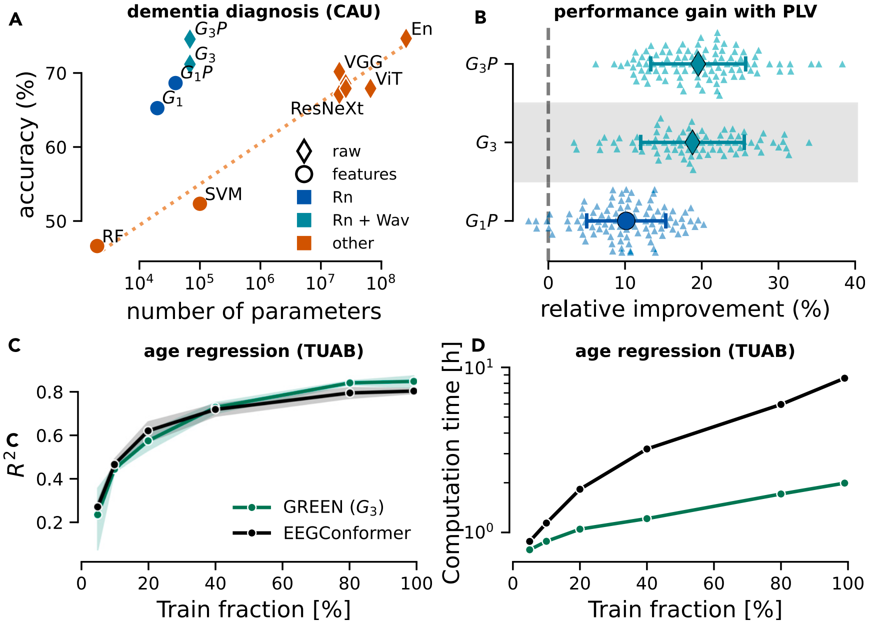

Figure 3. GREEN performance-complexity trade-off across components and extensions

(A) Model performance versus complexity measured with the number of parameters for the three-way classification of dementia diagnosis on the CAU dataset. The accuracy is measured on the benchmark from Kim and colleagues[15] using the same single train-test split. Results depicted in orange were replotted from the original publication. Results in blue show proposed models based on Riemannian geometry (Rn). Cyan represents the combination of Riemannian geometry and learned wavelets (Rn + Wav). Next to covariances, G_1P and G_3P also included pairwise phase-locking matrices to capture the phase information that might arise from the photic stimulation periods present in the EEG recordings from this dataset. It can be seen that the Riemannian models with learnable wavelets and explicit phase-locking features achieved high performance with orders-of-magnitude fewer parameters.

(B) Performance gain (error plot: SD) over the baseline model G_1 obtained by including phase-locking value (PLV) matrices in the pooling layer (G_1P), learnable wavelets (G_3), and the combination of both (G_3P). The same experimental protocol as in Figure 2 (100 shuffled splits, 20% test set size) was used. Including PLV matrices as a measure of phase synchrony in the pooling layer improved performance significantly for G_1P over G_1 and slightly for G_3P over G_3.

(C) Learning-curve comparison vs. EEGConformer architecture[68] on age prediction (TUAB). The G_3 model and the EEG conformer showed similar scaling behavior.

(D) Computational complexity vs. EEGConformer architecture on age prediction (TUAB). However, the G_3 model showed substantially slower increase in computation time, leading to an advantage higher than 400% as the full dataset was used (2 h for GREEN vs. 8.6 h for EEGConformer).

parametrization that log-linearly increases the spectral smoothing with frequencies and, therefore, suggests that a more precise localization in the frequency domain is beneficial for this task. As this task was performed across subjects, and alpha rhythms are known to show high individual variability, one plausible explanation could, therefore, be that G_3 might have used the low- and high-frequency wavelets as reference points to compute power ratios isolating alpha rhythms from their broadband background activity. Finally, inspecting the topography of the central wavelet after model training revealed a typical visual-occipital alpha pattern (Figure 5B). It is important to highlight that these visualizable EEG quantities were fully derived from the activations of the trained G_3 network without further external computation.

These results illustrate how the GREEN architecture can be practically used for biomarker exploration by extracting EEG descriptors such as power values and topographies that are well established for traditional statistical visualization and scientific hypothesis testing.

Exploration of GREEN for decoding of motor imagery from BCI EEG data

We developed GREEN with biomarker exploration as its primary context of use. However, both DL architectures and Riemannian models have been extensively studied and benchmarked for decoding of event-related EEG data in the context of BCIs. To explore the utility of GREEN for BCI applications and related ML research, we reproduced the MOABB (Mother of All BCI Benchmarks) BNCI2014_001 motor-imagery benchmark[73] as presented by Chevallier et al.[74] Across tasks and variants of generalization testing (within sessions, between session, between subjects), GREEN occupied high rankings with a median rank of 3 (Figure 6), which positions it favorably compared to other Riemannian models or other DL architectures. Inspection of specific benchmarks revealed that GREEN was among the highest-performing models for prediction within and between sessions (Figure S2). A more nuanced picture emerged for prediction across subjects. For the full four-class task, DL methods showed a marked advantage, whereas GREEN tended to perform better than other Riemannian methods (Figure S2, bottom left). For the two-class left/right problem, GREEN was on par with other DL models, showing a visible advantage over other Riemannian methods (Figure S2). We also explored the effect of constructing separate Riemannian representations per wavelet (G_3FB), in line with common spatial filter-bank methods, versus the default approach of GREEN (G_3) that constructs a single Riemannian representation from the covariance matrices concatenated across all frequencies (hence constructing a cross-frequency non-linearity). However, results were overall similar descriptively, showing higher average performance for G_3 on four out of six tasks (Figure S2). Taken together, these findings suggest that GREEN can be readily applied as a platform for ML research in BCI applications despite its primary design for the biomarker context of use.

DISCUSSION

The present work introduced a novel lightweight DL architecture termed GREEN (Gabor Riemann EEGNet) for predicting and

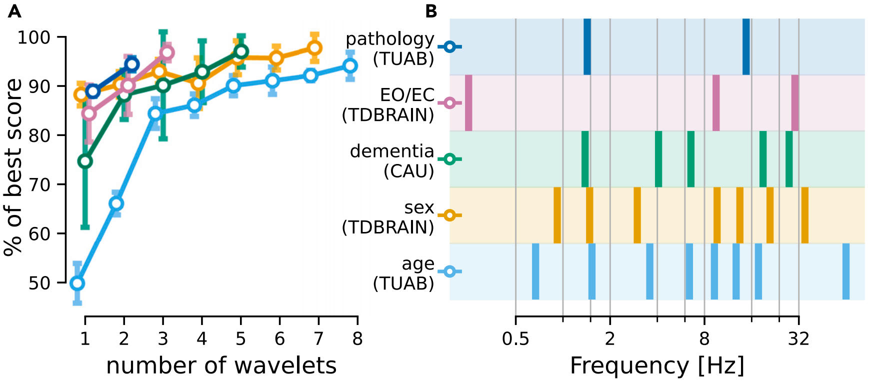

Figure 4. Optimal filter-bank composition depended on the prediction task

(A) Model performance as a function of model complexity in terms of numbers of wavelets (error plot: SD). Performance for all classification tasks and age regression (Figure 2) is presented as a percentage of the asymptotic value (best model) measured using a filter bank of 15 wavelets. Curves were plotted until the mean performance was less than one standard deviation away from the final asymptotic performance. Uncertainty estimates were obtained by repeating the analysis five times using random initializations. It can be seen that the number of wavelets required substantially varied across tasks and can be surprisingly low with two to three wavelets only (i.e., for pathology and EO/EC classification). By comparison, the baseline (cf. Table S1) used a filter bank of 49 wavelets.

(B) Frequencies of Gabor wavelets in the filter banks learned by the model for the different tasks. The number of wavelets was selected based on the learning curve presented in (A). Precise frequencies varied across tasks; however, low frequencies between 1 and 2 Hz and in the alpha range (8–12 Hz) were common choices.

learning from EEG in a unified framework. By integrating wavelets in a DL architecture, our approach provides a flexible and interoperable tool for probing modeling assumptions, exploring EEG data, and predicting with potential state-of-the-art performance. Furthermore, the relatively small number of parameters makes it easy to train this model with limited computation resources, hence fostering reproducibility and future research works.

A novel interoperable lightweight neural network for prediction of biomedical outcomes from EEG

We tested the GREEN model on five different prediction tasks and three datasets with EEGs from more than 5,000 human participants. GREEN uses convolution with parameterized Gabor wavelets, which presents a 2-fold benefit. First, they can easily be interpreted as band-pass filters and allow for the computation of features that have been extensively studied by the neuroscience community but may be overlooked by ML practitioners. Second, this parametrization is characterized by only two parameters, hence drastically limiting the total number of parameters in the architecture in comparison with other DL approaches,[15,68] which translated into substantial advantages in computation time. Furthermore, this architecture supports adequate geometric operations on the manifold of symmetric positive-definite (SPD) matrices, therein providing support for a broad set of data descriptors, including covariance, power-envelope correlation matrices, and other bi-variate interaction measures used in the literature for estimating functional connectivity in cortical networks.[35,75] The set of pooling layers is made even larger by the possibility of measuring cross-frequency interactions.[76,77] This was not possible using common

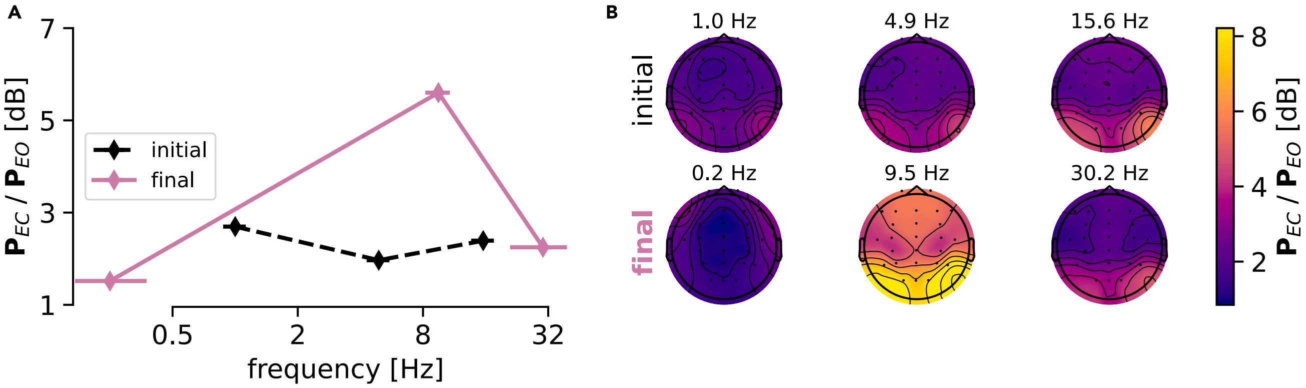

Figure 5. Task-specific learned representations provide actionable EEG quantities

(A) Averaged EEG power ratio (EC/EO) in dB derived from the wavelet transformed signal, using either the randomly initialized model (dashed line) versus trained (solid purple line). Horizontal error bars (SD) indicate the frequency-domain standard deviation of the wavelet. Vertical error bars (SD) indicate the spread in EEG power. The model focused on 8–12 Hz (typical location of occipital alpha activity with EC) as well as low and high frequencies as potential reference points. Markedly, after training, the frequency-domain standard deviation was minimal for the wavelet with the precise frequency near 9 Hz, which differs from commonly used parametrizations.

(B) Topographies for the three wavelets before (initial) and after (final) training. Powers obtained from the central wavelet showed an occipital topography characteristic for idling visual alpha rhythms. These power topographies were obtained from a forward pass (corresponding to the diagonal of the covariance matrix) and can be readily reused for secondary statistical analysis or visualization.

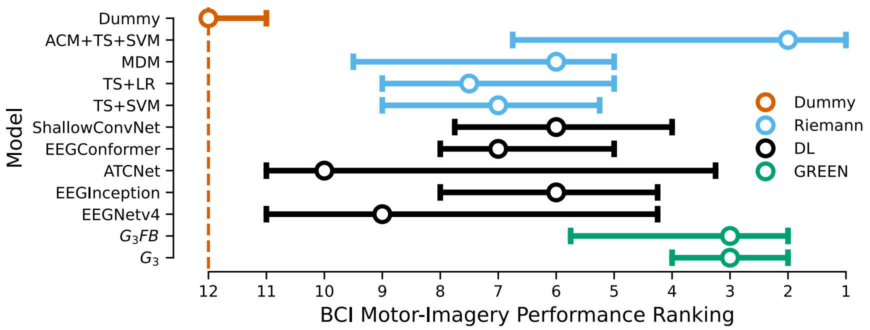

Figure 6. BCI performance ranking in MOABB benchmarks

Median performance ranking across MOABB motor imagery and movement benchmarks for purely Riemannian (light blue), convolutional deep architectures (black), and GREEN architectures. Error bars mark the inner 50% of the distribution. The G_3FB model uses frequency-specific pooling layers and frequency-specific Riemannian blocks, in line with classical filter-bank architectures. As before, $G3$ uses a single Riemannian representation across all frequencies, implying cross-frequency non-linearities. Overall, GREEN architectures performed favorably in occupying top rankings. For a detailed breakdown by task and prediction mode (e.g., within or between sessions), see Figure S2.

implementations of the Morlet parametrization, in which wavelets have varying temporal span depending on the frequency,[78] leading to discrepancies in the dimensions of the wavelet-transformed signals. While such terms typically vanish in the case of covariance matrices, the phase of the transformed could be removed by taking its modulus in order to compute the power-envelope correlation matrices. This new lightweight model shows state-of-the-art performances in established benchmarks such as age regression, sex prediction, or pathology classification with orders-of-magnitude fewer parameters than baseline DL architectures. Moreover, although initially designed for a context of use related to biomarker exploration, GREEN also showed strong potential for modeling event-related EEG as encountered in BCI applications.

A modular framework for testing modeling assumptions

Our work demonstrates how the modularity of DL can be used to express and investigate hypotheses about the underlying mechanisms relating EEG signals to biomedical outcomes. We designed the GREEN model to integrate established concepts from neuroscience and EEG (e.g., neural oscillations, power, and phase synchrony) with Riemannian geometry and DL methods through one single model. As the optimization engine is held constant across model comparisons, this approach provides high granularity for testing the impact of specific components.

Concretely, this has led us to the insight that certain tasks could be better solved by a few learned wavelets than a predefined grid of wavelets. Moreover, we found mixed evidence for the benefit of more complex non-linearities beyond the logarithm modeling assumption[1,34] as the added value of the FC layer was only visible for sex prediction and pathology decoding but not for the other prediction tasks. Finally, our approach facilitates the integration of additional feature computation, as illustrated by the addition of pairwise phase-locking value matrices, which we found to convey complementary information over the covariance for dementia diagnosis. At the methodological level, our work is related to recent non-deep Riemannian ensembling approaches in the BCI context, which combined different types of EEG connectivity features—cast into SPD matrices[79]—or stacked generalization[80] of sub-models for specific M/EEG features in the context of age prediction.[81,82] Another recent study proposed a Bayesian method extending the tradition of ICA for unsupervised learning of oscillatory components, resembling our work in providing inference on relevant frequencies.[83]

However, we would argue that one distinctive feature of our proposed framework is coherent computations using the same wavelet bases guided by one single supervised optimization objective. Taken together, the proposed framework supports a bottom-up approach to building DL models in which trusted components are gradually combined and tested rather than applying entire DL models developed in other domains (vision, speech) to EEG.

A potential workflow for EEG-biomarker exploration and practical considerations

The modular design and the choice of components mirroring concepts from the neuroscience literature have the potential to facilitate EEG-biomarker exploration in clinical applications. As one concrete toy example, we demonstrated for the well-studied Berger effect (closing eyes inducing 8–12 Hz oscillations) how frequency and EEG power can be directly extracted from a trained GREEN model via its forward pass. Although this example focused on a well-studied phenomenon, it allowed us to uncover surprising elements, such as the importance of low and high frequencies for detecting whether eyes are closed or open. Interestingly, inspection of the frequency standard deviation of the wavelets revealed a non-monotonic function with a more localized wavelet in the expected alpha-band range around 10 Hz and broader frequency spread for wavelets located at low and high frequencies. It is noteworthy that this type of pattern reflects a unique capability of the proposed architecture to learn a flexible filter-bank parametrization that is not bound to traditional rules such as increasing the frequency standard deviation log-linearly with increasing frequencies as, e.g., Morlet wavelets.[5,35,67,78] Such deviations can be scientifically exploited as they convey insights into the data.

To continue with this example, we found the consistent choice (across five random initializations) of the 0.25 Hz low-frequency wavelet to be surprising as the data were high-pass filtered at 1 Hz. Additional inspection of the intermediate representations of an individual EEG recording Figure S1 revealed that, as expected given the filtering, the amplitude of the 0.25 Hz wavelet-transformed signal was an order of magnitude lower. Surprisingly, we observed physiological oscillatory activity,

pointing at information in low frequencies that was relevant for prediction and therefore recovered by the model from the attenuation of the high-pass filter.

These observations lead us to argue that representations learned by the GREEN architecture facilitate scientific exploration by readily providing established EEG descriptors such as power estimates that encourage visual and statistical comparisons. This practical level of interpretability is in particular important given the regulatory constraints on statistical analysis of biomarkers. These require that biomarkers can be reproducibly measured, which, e.g., in a large multi-centric clinical trial, is operationally more likely to succeed with simpler procedures. Furthermore, biomarkers must stand in a mechanistic relationship to the disease biology and targeted physiological pathways, which has been repeatedly established for EEG power in specific examples.[10,84–86] In this context, ML could assist EEG-biomarker development through at least two main routes, which we stylize for clarity: route A uses learned representations to generate novel insights into the data and inform an explicit rule-based, operational biomarker recipe. Route B directly uses model predictions or learned representation as biomarker values. Importantly, the GREEN architecture provides high flexibility for pursuing any of these routes. However, what makes it different is that its representations have names in the neuroscience literature and that its lean design facilitates rapid exploration cycles of model training and subsequent visual or statistical exploration. We argue that these properties should largely improve the capacity of GREEN to support route A while its high performance as a prediction engine maintain access to route B without compromises.

This brings us to one more consideration. In our benchmarks, GREEN revealed its potential for high prediction capacity enabled by structural priors and constraints on computational complexity. It is fast to train and therefore supports scientific agility, not only translating into more exploration in less time and for lower electricity costs[61,62] but also removing obstacles for rigorous statistical inference practices based on resampling procedures. The motivation for the design of GREEN was to combine the potentially higher performance and expressiveness of DL models with the agility and speed of simpler shallow models. We see this as an important property for a scientific tool in empirical disciplines dealing with high-dimensional data (such as neuroscience) in which core discoveries are often driven by serendipitous exploration. A lean, efficient, and scientifically interoperable modeling pipeline contributes to the democratization of computation.[87,88]

Finally, the GREEN framework should be seen as a complementary tool rather than a drop-in replacement for previously proposed wavelet toolboxes.[67] Indeed, learning filter banks in a supervised way can be a starting point for further analyses. A second step might consist in using a more fine-grained grid in the identified frequency ranges or computing additional features from the wavelet-transformed signal. Another approach that is presented in this work is to use predefined filter banks as an initialization of the convolution layer and assess to what extent the model deviates during training.

Theoretical considerations

We wish to point out that, beyond these practical applications, the proposed GREEN framework possesses favorable properties for future method development in mathematical modeling to study data-generating mechanisms. First of all, GREEN performs operations that are consistent with prediction models that benefit from desirable theoretical properties that were investigated formally in prior work.[34,89] This is eminently true for G_1, which was designed to reproduce previously proposed pipelines. For this model, prior work[89] showed that it enjoys the property of statistical consistency under the assumption that the outcome is generated by a linear combination of the log power of stationary EEG sources in the presence of linear mixing between sources and electrodes. Notably, the theoretical analysis from Sabbagh and colleagues[89] only treats the spatial dimension of the problem and assumes some arbitrary band-pass filter; hence, there is no loss of generality by the introduction of learnable wavelets. Furthermore, we would argue that the introduction of additional hidden layers in ($G_2 - G_3$) preserves this theoretical analysis. In fact, we found limited evidence that the hidden layers alone led to changes in the models' performance (Figure 2).

This brings us to a final consideration. Despite added complexity and expressiveness, the SPD representations learned in pooling layers and BiMaps can be reused with non-deep approaches, including as inputs for methods from the rich literature of kernel methods,[90] which can accommodate manifold-valued data,[91] hence enabling various applications in high-dimensional statistical learning.[92–94] These properties might facilitate future work on developing uncertainty estimation and statistical inference within the GREEN framework.

Limitations and future work

GREEN unlocks performance with a lean architecture design enabled by the choice of strong structural priors such as sinusoidal complex wavelets and Riemannian geometry. This leads to characteristic inductive biases that allow sparse representations but may hit limitations. For instance, the ability of the wavelet convolutions to capture complex neural oscillations (e.g., documented cases of cross-frequency couplings, or non-sinusoidal or asymmetric waveforms,[76,95,96]) with a reasonably sized filter bank of wavelets requires experimental testing. We identified three potential extensions of the present work to tackle this challenging task. First, to capture and study non-sinusoidal waveforms, the filter bank could be constrained to contain groups of harmonic wavelets. Second, in an attempt to capture cross-frequency correlations, the pooling layers could also be extended to compute power-envelope covariances. Similarly, measures of phase-amplitude coupling could be considered. Another direction would be to iterate wavelet convolutions and modulus operator similarly to a scattering network.[97] Such approaches are known to capture complex patterns, allowing discrimination between different signals that share a similar power spectrum. However, they typically use a fixed grid of wavelets, and using a cascade of convolution would hinder the explainability of the model. Finally, this work studied Gabor wavelets due to their theoretically desirable properties; however, other waveforms from the rich wavelet literature[98] that support such parametrization might as well be explored. In addition, while covariance matrices are useful for encapsulating the overall correlation structure between different channels over time windows, such representations are inherently limited in their ability to capture transient patterns.

Non-stationary activities are, however, known to carry physiological information associated with certain conditions.[99,100] Mitigation strategies include working with distributions, instead of single covariance matrices, by using methods from the optimal transport framework[101] or using sequences of inputs along with recurrent neural networks[102] or attention mechanisms.[103]

This architecture enabled practical interpretability by allowing to carefully gauge the impact of network components on prediction performance. However, this is fundamentally different from statistical inference or variable-importance estimation.[104–106] Recent extensions of permutation-based importance hold promise to provide statistical error control and effective variable-importance detection in the context of DL models and high-dimensional correlated inputs.[107–109] The successful application of high-dimensional inference procedures will be an important opportunity for future work with the potential to add statistical rigor to the practical inference provided by the structural design choices of GREEN.

Finally, our proposed framework is yet to be explored in the context of interventional studies to address specific medical hypotheses or questions. Finding frequency bands and spatial patterns that are predictive of various endpoints, such as treatment response, disease progression, or adverse events, remains a high-priority task for future applied work to inform biomarker development. In this context, self-supervised learning[110,111] could be a promising extension of GREEN to tackle the unsolved challenge of cross-dataset transfer, for instance across centers. Even though EEG datasets from the general population keep increasing, for instance when considering advances brought by innovative multi-site consortia[112–114] in the dementia space, the number of patients included in clinical trials remains limited. Self-supervised approaches could, therefore, be an effective strategy for learning clinically robust representations that can be fine-tuned to solve dataset-specific tasks and, at the same time, cope with covariate shifts.[115,116] Concretely, the GREEN architecture could be used as the encoder of more complex self-supervised learning architectures.[111,117] As final limitation for clinical application, we have not studied the capacity of our model to adjust to high-density EEGs with 128 or more channels. How the model would predict from such data without modification and what would be the optimal size of the filter banks remain interesting open questions.

Conclusions

The new GREEN architecture was designed to facilitate the work of researchers working on biomarker technologies and clinical applications with EEG. Our results suggest that this effort to incorporate neuroscientific domain knowledge into the architecture design has the potential to offer new angles for approaching clinical problems. At the same time, this work is an interdisciplinary attempt to bridge biostatistical thinking in the context of clinical studies with ML research. The design philosophy of GREEN reveals its own vision of DL for EEG in the era of large-scale models: improving the effectiveness of well-known EEG signatures such as EEG power and phase synchrony through strategic architecture design rather than perpetually building larger models following the "bigger-is-better" paradigm. To support this effort, we share the source code of GREEN (https://github.com/Roche/neuro-green).

METHODS

Related work

Published DL architectures for EEG focus on end-to-end processing of raw EEG data[15,47,118] and provide state-of-the-art prediction performance on a range of tasks. However, the scaling in terms of the number of parameters of such models seems unsustainable given the scarcity of EEG data, and their lack of interpretability hinders their impact for biomarker discovery.[61] In this background review, we focus on classical spectral analysis tools and Riemannian geometry to motivate architecture design choices for our work that aims at overcoming limitations of current DL applications to EEG.

Spectral analysis of EEG recordings is a key step for identifying clinically relevant features. However, the classical Fourier transform is not always well suited for such data since it lacks temporal resolution. Time-resolved methods such as the complex wavelet transform are often preferred as they not only capture temporal variations of the frequency content but also allow for frequency-dependent spectral smoothing. This work will focus on the wavelets introduced by Dennis Gabor, which present an optimal trade-off between time and frequency localization.[119] A Gabor wavelet, ϕ_f, consists of a complex sinusoid of frequency *f* modulated by a Gaussian window characterized by its standard deviation σ_t,

$$\phi_f(t) = \frac{1}{\sqrt{2\pi}\sigma_t} \exp\left(\frac{-t^2}{2\sigma_t^2}\right) \exp(2i\pi f t). \qquad \text{(Equation 1)}$$

Like many natural processes, the power of EEG signals have a $1/f$ scaling.[1] To analyze such signals, wavelet families are constructed using the logarithmic frequency scale in octaves introduced by Jean Morlet.[78] This provides a non-orthogonal basis of wavelets that supports a wide array of techniques for studying brain function and can easily be interpreted as band-pass filters. In such parametrizations, wavelets are more localized in time and more spread in the frequency domain at high frequencies. This parametrization is therefore helpful for comparing brain activity along the frequency spectrum by providing physiologically appropriate smoothing (σ_t log linearly decreasing with *f*), which facilitates visualization and biomarker exploration.[5,9–11,84]

In practice, neuroscientists use large filter banks to capture the entire frequency content of the signal. From a theoretical perspective, this does not lead to optimal wavelet bases in the sense of a minimally sized complete representation of the signal,[98] and, as a result, Morlet filter-bank features are often non-sparse and highly correlated. Combining such Morlet-parametrized wavelets and ridge regularization[120] proved effective in a recent study where the authors observed that prediction performance saturated at a certain filter-bank size but did not degrade.[67]

While proving that this approach works well for regularized linear models, such non-sparse representations can lead to intractable computation costs for complex model architectures. To mitigate this issue, the parameters of the wavelet can be learned in a neural network in order to replace handcrafted filter banks. This approach was followed for speech recognition[121] from audio data, which we did not consider here as it is designed for 1-d signals instead of spatially mixed multichannel signals as

in EEG. The only application of Gabor wavelets to EEG relied on real-valued wavelets, which provides incomplete representation (e.g., hindering the explicit computation of phase-related features).[54] Moreover, real-valued wavelets lack the desirable property of being invariant to small shifts after taking the modulus. Theoretically, this can translate to smaller activation values, hence requiring a larger number of windows or data points. As pointed out by Mike X. Cohen,[122] this is circumvented by using complex-valued wavelets. Regarding prediction performance, results reported in Barmpas et al.[54] suggest that real-valued wavelets lead to performance comparable to other deep convolutional architectures on BCI tasks. Concerning interpretability, the BrainWave-Scattering Net architecture does not explicitly compute power values although the combination of scattering transforms, and FC layers should be able to implicitly represent EEG power. The authors inspected the model in terms of the spatial filter weights.

Riemannian geometry is a theoretical framework for analyzing SPD matrices that forms the foundation of robust ML methods extensively applied to various prediction tasks involving EEG signals. These methods mostly focus on the processing of sample covariance matrices, which up to some regularization are SPD.[42,43,89,123] Such approaches present two major assets. First, they utilize an affine invariant metric, which is relevant for EEG as the data-generating process can be modeled as a linear mixing of sources.[31,32,34] Second, they project SPD matrices to a Euclidean tangent space, therefore enabling the use of classical statistical learning tools.[124] Importantly, the tangent-space mapping involves matrix logarithms that match well the dominance of lognormal distributions in brain structure and function.[1] In practice, the log-Euclidean metric is often preferred to the affine invariant due to its superior computational efficiency while preserving theoretically desirable properties and yielding similar experimental results.[125] The tangent-space vector of an SPD matrix $\mathbf{X}$ is obtained by applying the logarithm map $\phi^{LE}_{X_{ref}}$ at the reference point $\mathbf{X}_{ref}$,

$$\phi^{LE}_{X_{ref}}(\mathbf{X}) = vect(Log(\mathbf{X}) - Log(\mathbf{X}_{ref})), \qquad \text{(Equation 2)}$$

where Log is the matrix logarithm and vect is the vectorization operator that stacks the upper triangular elements of a matrix and multiplies the off-diagonal terms by $\sqrt{2}$.

Geometric operations for processing SPD matrices have recently been integrated into deep neural networks with mostly four operations[64,126]: the bilinear mapping, the rectified eigenvalue, the logarithm of eigenvalues, and the batch normalization. Nevertheless, when applied to EEG signals, these operations present two main limitations that we aim to address in this work. First, when processing sample covariance matrices in the context of EEG, a well-documented hurdle is the rank deficiency induced by EEG preprocessing.[89,127] This issue is often ignored or *ad hoc* mitigation approaches are used, such as selecting a limited number of channels or adding a scalar to diagonal terms.[55,56] Second, the projection to tangent space requires a reference point ($\mathbf{X}_{ref}$ in Equation 2), for which the mean across subjects is an intuitive choice as it reveals high-amplitude, spatially structured noise related to volume conduction and anatomy-driven signal mixing. Intuitively, this allows computing power ratios relative to background activity.[24,34]

One can note that, in DL models, logarithm maps use the identity matrix as reference rather than the average. When setting the reference to identity, the log map operation simplifies to the matrix logarithm, which reduces the computational and engineering complexity. However, this may not be optimal for EEG applications as the spatial patterns captured by between-electrode covariance matrices are governed by structured noise reflecting volume conduction.[128] Incorporating biophysically meaningful reference points such as the average covariance therefore enables explicit signal denoising. Learning the reference point was, therefore, an important step for matching the mathematical operations and the performance of non-deep Riemannian models.[34,67]

Taken together, the relevance and successful application of wavelets for spectral analysis and Riemannian geometry for prediction task suggest integrating these elements for neuroscience-informed DL architectures.

Model

In the equations presented in following paragraphs, $\mathbf{X}$ (with additional upper or lower scripts and dimension specification) is used to describe the input of different layers but does not refer to EEG signals.

Parametrized convolution

The first block of this architecture is the parametrized convolution layer. The kernel consists of complex-valued Gabor wavelets (Equation 1) in which the frequency of the carrier (f) and the standard deviation of the Gaussian window (σ_t) are learned parameters. These time-frequency atoms can be interpreted as band-pass filters. As shown in Figure 1, for each sample, multiple time windows (E) are processed by this layer. Preliminary experiments showed that using $T = 10$-s time windows led to good results in the explored range. For the initialization of the parameters f and σ_t, we used the Morlet parametrization described above unless explicitly stated that a random initialization was used.

Pooling layer

The wavelet-transformed signal is then passed through a pooling layer in order to compute feature maps. In the basic configuration, the pooling layer includes the sample covariance matrix computed from the wavelet-transformed signals. Concretely, given two wavelet-transformed signals $\mathbf{X}_i, \mathbf{X}_j \in \mathbb{C}^{C \times T}$ referring to wavelets i^{th} and j^{th} of the filter bank, the covariance matrix is

$$\mathbf{C}_{i,j} = \frac{1}{T} Re\left(\mathbf{X}_i \mathbf{X}_j^{\top}\right) \qquad \text{(Equation 3)}$$

This leads to a matrix in which diagonal blocks contain the interactions between channels at a given frequency $\mathbf{C}_{i,i}$, whereas off-diagonal blocks contain cross-frequency interactions between channels, $\mathbf{C}_{i,j}$. Compared to other work using real-valued Gabor wavelets,[54] this approach has advantages for interpretation as the diagonal values of this matrix represent classical EEG power that can be readily visualized or reused for statistical exploration. Of note, in the present setting, the cross-frequency covariances represented by off-diagonal blocks can be expected to vanish. While we did not systematically explore this architecture component in the present work, this opens various opportunities for designing pooling layers for cross-frequency coupling.[76,77]

The complex wavelet-transformed signal allows for the computation of other features such as the pairwise phase-locking value.[59] The pairwise PLV matrix **P** contains the elements

$$[\mathbf{P}]_{k,l} = \frac{1}{T}\left|\sum_{t=1}^{T} \exp\left(i\left(\frac{x^k(t)}{|x^k(t)|} - \frac{x^l(t)}{|x^l(t)|}\right)\right)\right|, \quad \text{(Equation 4)}$$

where (k, l) is a pair of sensors and $x^k(t)$ is the instantaneous value of the wavelet-transformed signal. Dividing by the respective modulus provides a measure of the instantaneous phase difference, which is then averaged over time to compute the PLV. Besides these two examples of covariance and phase locking that we study in the Results, other features such as the power-envelope correlation matrix[9] or coherence[60] and various measures of cross-frequency relationships (e.g., phase-phase, phase-amplitude, amplitude-amplitude) could be used. Furthermore, the framework is applicable to the analysis of event-related brain activities if the EEG datasets subjected to the analysis are selected in an event-related manner (for example, decoding of movement intentions in the context of BCIs).

Shrinkage layer

In the finite sample regime, sample covariance matrices are known to overestimate the range of eigenvalues, which sometimes lead to numerical instabilities.[63,129] To mitigate this issue, we built on top of prior work on covariance shrinkage operators, which are known to outperform *ad hoc* approaches, especially in the EEG context.[128] Here, we implemented a shrinkage layer, which given a matrix $\mathbf{X} \in \mathbb{R}^{p \times p}$ performs the operation

$$\mathbf{X}_{shrunk} = (1-\alpha)\cdot\mathbf{X} + \alpha\frac{Tr(\mathbf{X})}{p}\cdot\mathbf{X}, \quad \text{(Equation 5)}$$

where the strength of the shrinkage α is a learned parameter. This operator presents the desirable property of preserving the trace of the matrix. For covariance matrices, this trace is the total power, which is a critical feature of EEG signals often used as such in ML pipelines.

BiMap layer

In the context of EEG data, covariance matrices can be rank deficient due to preprocessing and artifact removal[67] and therefore do not belong to the SPD manifold. To mitigate this issue,[89] we formally proved that projecting such matrices to a common subspace enables regression modeling on SPD manifolds with statistical guarantees despite rank-deficient covariance inputs. To incorporate these insights, the subspace projection can be implemented using a BiMap layer, which given an input matrix $\mathbf{X} \in \mathbb{R}^{C \times C}$, performs the operation

$$BiMap(\mathbf{X}) = \mathbf{W}\mathbf{X}\mathbf{W}^{\top}, \quad \text{(Equation 6)}$$

where the weight matrix $\mathbf{W} \in \mathbb{R}^{Q \times C}$ is constrained to be semi-orthogonal,[64] using a manifold optimization algorithm[130] (for more details, see Note S1). This step is consistent with classical spatial filtering.[33,41,131] Empirical exploration of this approach confirmed that the eigenvalues of the projected feature maps were full rank. Importantly, we found that this approach was also more robust to rank deficiencies induced by preprocessing steps such as independent component analysis,[132] which is practical as that approach allows us to avoid the search for the smallest common subspace.[67,89] Furthermore, we observed that not constraining weight matrices to be semi-orthogonal still led to mappings that ensured the strict positivity of the resulting features maps, hence fully supporting Riemannian computation. Therefore, to make computation more efficient, unless specified, we did not use a constrained optimization algorithm for the weights of the BiMap layer.

Combined ReEig and LogMap layer

The rectified eigenvalue (ReEig) layer creates a non-linearity transforming eigenvalues. Besides, this operation forces the eigenvalues to be greater than a given threshold, therefore ensuring numerical stability during training. Finally, the logarithm map (LogMap; see Equation 2), initially introduced as LogEig, uses the log-Euclidean metric, which empirically provides similar results to the affine invariant metric at a cheaper computation cost.[125] However, we differentiate our approach from others that use the identity matrix as reference. Instead, we use a running log-Euclidean mean $\mathbf{X}_{ref}$, which preserves the consistency with the Riemannian distances.[133] The operation is provided in Equation 2. The update rule of the running mean at step k is given by

$$\mathbf{X}_{ref}^{k} \leftarrow Exp\left((1-\theta)\cdot Log(\overline{\mathbf{X}}_B) + \theta \cdot Log\left(\mathbf{X}_{ref}^{k-1}\right)\right), \quad \text{(Equation 7)}$$

where θ is the momentum and $\overline{\mathbf{X}}_B$ is the log-Euclidean mean over the current batch. *Log* and *Exp* respectively correspond to the matrix logarithm and exponential. Other works also explored Riemannian batch normalization but we did not observe any benefit of such a layer in our experimental setting in our past work.[126] To avoid redundant computation, we merged the last ReEig and LogMap computations by rectifying the eigenvalues and computing their logarithm in a single diagonalization step.

FC layer

Finally, a classical multi-layer perceptron head can be added along other DL layers. Initial hyperparameter exploration led to good results with two FC layers (64 and 32 hidden units) with batch normalization and dropout ($p = 0.333$) and a Gaussian error linear units (GELU) non-linearity.[134] For regression and classification tasks, we respectively used the mean-square-error and the cross-entropy loss functions.

Datasets and preprocessing

We used three public datasets with institutionally controlled access. Our focus was on subject-level prediction using resting-state EEG, which is most commonly used in clinical settings and biomarker applications. Single biomedical outcomes of human participants are predicted from the corresponding EEG recordings, and the training and testing sets form a population of participants. This setup differs from the popular event-level prediction from event-related activity, which is used in BCI where outcomes are defined for specific events (e.g., stimuli or behavior).

We therefore considered larger biomedical EEG datasets (more than 1,000 participants) with published benchmarks for deep and non-deep ML models across diverse regression and classification problems. Finally, the selection of datasets covers a heterogeneous population, with healthy participants and patients suffering from psychiatric or neurodegenerative disorders, hence allowing us to gauge the versatility of our approach.

TUAB

The TUAB dataset contains resting-state EEG recordings from 2,993 patients with normal and abnormal labels provided by medical experts.[16,135] The recordings were acquired using variable numbers of electrodes (24–36). We used only the 21 channels that were common to all subjects and conformed to the 10–20 configuration. For the pathology classification task, we used all subjects and, for the age-prediction task, we considered a subset of 1,278 healthy subjects to obtain results comparable with those published in previous works.[66,136] Similarly to our previous work,[67] we cropped recordings to a common duration of 15 min.

TDBRAIN

The TDBRAIN database contains 1,274 EEG recordings of patients with psychiatric conditions.[14] All recordings have been acquired using 26 electrodes positioned according to the 10-10 international system. Annotations indicated segments in which the subjects had their eyes open or closed are also provided. The dataset also contains demographic information including the sex of the patient (620 female, 654 male).

CAU

The CAU Hospital dataset contains recordings from 1,155 patients along with clinical diagnoses.[15] Subjects were grouped into three categories: normal, MCI, and dementia. The recordings were acquired using 19 electrodes positioned according to the international 10–20 system. It should be noted that this dataset includes resting state but also EEG recorded during photic stimulation exposure periods. The average and standard deviation of the recording duration are respectively 13.34 and 2.83 min.

Common processing steps

For all the datasets, the preprocessing included average referencing and band-pass filtering between 1 and 100 Hz. High-amplitude artifacts were removed using the autoreject algorithm.[137] TUAB and TDBRAIN were resampled to 125 Hz and CAU to 140 Hz (half of the sampling frequency) to reduce the memory footprint. For the eyes-open versus eyes-closed classification task, we additionally included an ICA (independent component analysis) step to remove ocular artifacts that would otherwise be trivially predictive. We adopted the ICA pipeline as described in our work[67] based on the fast PICARD (Preconditioned ICA for Real Data) implementation[138] of the FastICA algorithm[132] and the ICLABEL method[139,140] for detection of artifact components. For all datasets and tasks, each sample consisted of 10 windows ($E = 10$) of 10 s ($T = 10\,s$) randomly drawn from the recordings.

Additional BCI motor-imagery benchmark

To position our model within the field of ML research for BCI, we reproduced MOABB benchmarks,[74] focusing on the popular motor-imagery benchmark BNCI2014_001.[73] We considered two tasks: four-way classification task between left hand, right hand, tongue, and feet motor imagery and binary classification between left and right motor imagery. We reported the performances of the different models using three different evaluation methods: within session, between session, and between subject. The benchmark was implemented using the Benchopt package (https://benchopt.github.io/) to allow reproducible experiments.

Baseline model

In previous publications, methods based on Riemannian geometry have been reported to perform similarly to a wide range of DL architectures on the pathology classification task on TUAB[141] and provided comparable results to a DL model on the age- and sex-prediction tasks on TUAB and TDBRAIN.[34,66,67] Here, we focused on the model presented by Bomatter and colleagues, which we call baseline throughout this work. The GREEN architecture was directly inspired by this baseline, capitalizing on wavelets and incorporating equivalent operations in its shrinkage and BiMap and LogMap layers. Choosing this baseline allowed us to understand which precise additional computational steps and non-linearities can add improvements.

The baseline used features extracted from wavelet-transformed signals using a predefined Morlet filter bank (49 wavelets with central frequencies logarithmically spaced between 1 and 64 Hz). Sample covariance matrices were then estimated and projected to the SPD manifold using principal-component analysis (PCA) to account for their rank deficiency.[89] Finally, a projection to tangent space (Equation 2) followed by a linear model with ridge regularization were applied.[120] As in the original work, we used the scikit-learn[142] implementation RidgeCV, which uses generalized cross validation[143] for hyperparameter selection of the penalization strength. We tested 100 values between 10^{-5} and 10^5 on a logarithmic grid.

Additional models

CAU dataset

To compare results obtained using other DL architecture on the CAU dataset, we reproduced the exact train-test splits and reported the previously published benchmarks.[15]

TUAB dataset

To make comparisons to more recent deep convolutional transformer architectures, we also included the EEGConformer model,[68] which should provide capacity for higher complexity than GREEN.

BCI benchmark

From all available model included in the Benchopt BCI benchmarks, we focused on four popular DL models and four popular Riemannian models that showed high performance after initial testing: ATCNET,[144] EEGNet,[48] EEGInception,[145] and ShallowNet[47]; and ACM + TS + SVM,[146] MDM,[147] TS + LR,[148] and TS + SVM.[148] To make comparisons to more recent deep convolutional transformer architectures, we also added the EEGConformer model[68] to the benchmark. For all DL models, we used the default parameters provided in the publicly available implementations and did not perform any hyperparameter tuning. We trained DL models for 300 epochs using early stopping, based on a validation set representing 20% of the training set, with a patience of 100 epochs. For non-deep Riemannian models, the hyperparameter tuning was used as described by the authors in the respective initial publications. Although the previous work focused on the within-session evaluation,[74] we computed the results on all three modes of prediction to provide broader comparisons.

Statistical inference for model comparisons

Except for the pathology classification on CAU where the test set from the original publication was used for comparison (Figure 3A), we used a Monte-Carlo cross-validation scheme to compare the performance of the different models. The experimental protocol consist of 100 random splits with a test set

containing 20% of the data as described by Bouckaert and Frank.[149] To make up for the underestimation of the variance in cross-validation settings, we followed Nadeau's and Bengio's corrected resampled t test[65]

$$t = \frac{\frac{1}{K}\sum_{i=1}^{K} x_i}{\sqrt{\frac{1}{K}+\frac{n_{test}}{n_{train}}\hat{\sigma}^2}},$$

where K is the number of splits, n_{train} and n_{test} are respectively the train and test sizes, x_i is the performance difference between the two models, and $\hat{\sigma} = \frac{1}{K-1}\sum_{i=1}^{K}(x_i - m)$ is the naive estimate of the variance, m being the mean performance difference.

RESOURCE AVAILABILITY

Lead contact

Further information and requests for resources should be directed to the lead contact for questions related to resources, Joseph Paillard (joseph.paillard@roche.com).

Materials availability

No new biological materials were generated by this study.

Data and code availability

Our source code is available at GitHub (https://github.com/Roche/neuro-green) and has been archived at Zenodo.[150]

ACKNOWLEDGMENTS

We thank Bruno Aristimunha Pinto for his valuable feedback on the previous preprint version of our work, allowing us to reproduce the BCI benchmark and his code review when adding the GREEN architecture to the benchmark.

We thank our colleagues Eric Keller and Rasmus Iten for their valuable guidance on open-sourcing and licensing our code and software.

AUTHOR CONTRIBUTIONS

Conceptualization, D.A.E., J.F.H., and J.P.; data curation, D.A.E. and J.P.; formal analysis, D.A.E., J.F.H., and J.P.; investigation, J.P.; methodology, D.A.E. and J.P.; project administration, D.A.E.; software, J.P.; supervision, D.A.E.; validation, D.A.E. and J.P.; visualization, D.A.E. and J.P.; writing – original draft, D.A.E. and J.P.; writing – review & editing, D.A.E., J.F.H., and J.P.

DECLARATION OF INTERESTS

J.P., J.F.H., and D.A.E. are full-time employees of F. Hoffmann - La Roche Ltd.

SUPPLEMENTAL INFORMATION

Supplemental information can be found online at https://doi.org/10.1016/j.patter.2025.101182.

Received: July 24, 2024
Revised: November 14, 2024
Accepted: January 21, 2025
Published: February 13, 2025

REFERENCES

1. Buzsáki, G., and Mizuseki, K. (2014). The Log-Dynamic Brain: How Skewed Distributions Affect Network Operations. Nat. Rev. Neurosci. *15*, 264–278. https://doi.org/10.1038/nrn3687.
2. Dement, W., and Kleitman, N. (1957). Cyclic variations in EEG during sleep and their relation to eye movements, body motility, and dreaming. Electroencephalogr. Clin. Neurophysiol. *9*, 673–690.
3. Rampil, I.J. (1998). A primer for EEG signal processing in anesthesia. The Journal of the American Society of Anesthesiologists *89*, 980–1002.
4. Jasper, H.H. (1949). Electrical signs of epileptic discharge. Electroencephalogr. Clin. Neurophysiol. *1*, 11–18.
5. Tallon-Baudry, C., Bertrand, O., Delpuech, C., and Pernier, J. (1996). Stimulus Specificity of Phase-Locked and Non-Phase-Locked 40 Hz Visual Responses in Human. J. Neurosci. *16*, 4240–4249. https://doi.org/10.1523/jneurosci.16-13-04240.1996.
6. Klimesch, W., Doppelmayr, M., Russegger, H., Pachinger, T., and Schwaiger, J. (1998). Induced alpha band power changes in the human EEG and attention. Neurosci. Lett. *244*, 73–76.
7. Polich, J. (2007). Updating P300: An integrative theory of p3a and p3b. Clin. Neurophysiol. *118*, 2128–2148.
8. Bekinschtein, T.A., Dehaene, S., Rohaut, B., Tadel, F., Cohen, L., and Naccache, L. (2009). Neural signature of the conscious processing of auditory regularities. Proc. Natl. Acad. Sci. USA *106*, 1672–1677.
9. Hipp, J.F., Engel, A.K., and Siegel, M. (2011). Oscillatory synchronization in large-scale cortical networks predicts perception. Neuron *69*, 387–396.
10. Frohlich, J., Miller, M.T., Bird, L.M., Garces, P., Purtell, H., Hoener, M.C., Philpot, B.D., Sidorov, M.S., Tan, W.-H., Hernandez, M.-C., et al. (2019a). Electrophysiological phenotype in angelman syndrome differs between genotypes. Biol. Psychiatr. *85*, 752–759. https://doi.org/10.1016/j.biopsych.2019.01.008.
11. Hawellek, D.J., Garces, P., Meghdadi, A.H., Waninger, S., Smith, A., Manchester, M., Schobel, S.A., and Hipp, J.F. (2022). Changes in brain activity with tominersen in early-manifest Huntington's disease. Brain Commun. *4*, fcac149. https://doi.org/10.1093/braincomms/fcac149.
12. Casson, A.J. (2019). Wearable EEG and beyond. Biomed. Eng. Lett. *9*, 53–71.
13. Fiedler, P., Fonseca, C., Supriyanto, E., Zanow, F., and Haueisen, J. (2022). A high-density 256-channel cap for dry electroencephalography. Hum. Brain Mapp. *43*, 1295–1308.
14. van Dijk, H., van Wingen, G., Denys, D., Olbrich, S., van Ruth, R., and Arns, M. (2022). The Two Decades Brainclinics Research Archive for Insights in Neurophysiology (TDBRAIN) Database. Sci. Data *9*, 333. https://doi.org/10.1038/s41597-022-01409-z.
15. Kim, M.-j., Youn, Y.C., and Paik, J. (2023). Deep Learning-Based EEG Analysis to Classify Normal, Mild Cognitive Impairment, and Dementia: Algorithms and Dataset. Neuroimage *272*, 120054. https://doi.org/10.1016/j.neuroimage.2023.120054.
16. Obeid, I., and Picone, J. (2016). The Temple University Hospital EEG Data Corpus. Front. Neurosci. *10*, 196.
17. Delorme, A., and Makeig, S. (2004). EEGLAB: An open source toolbox for analysis of single-trial EEG dynamics including independent component analysis. J. Neurosci. Methods *134*, 9–21.
18. Benca, R.M., Obermeyer, W.H., Larson, C.L., Yun, B., Dolski, I., Kleist, K.D., Weber, S.M., and Davidson, R.J. (1999). EEG alpha power and alpha power asymmetry in sleep and wakefulness. Psychophysiology *36*, 430–436.
19. Dressler, O., Schneider, G., Stockmanns, G., and Kochs, E.F. (2004). Awareness and the EEG power spectrum: Analysis of frequencies. Br. J. Anaesth. *93*, 806–809.
20. Otto, K.A. (2008). EEG power spectrum analysis for monitoring depth of anaesthesia during experimental surgery. Lab. Anim. *42*, 45–61.
21. Buzsáki, G., Logothetis, N., and Singer, W. (2013). Scaling brain size, keeping timing: Evolutionary preservation of brain rhythms. Neuron *80*, 751–764.
22. Bedard, C., Kroeger, H., and Destexhe, A. (2006). Does the 1/f frequency scaling of brain signals reflect self-organized critical states? Phys. Rev. Lett. *97*, 118102.

CellPress
OPEN ACCESS

23. Pritchard, W.S. (1992). The brain in fractal time: 1/f-like power spectrum scaling of the human electroencephalogram. Int. J. Neurosci. *66*, 119–129.

24. Nikulin, V.V., Nolte, G., and Curio, G. (2011). A novel method for reliable and fast extraction of neuronal EEG/MEG oscillations on the basis of spatio-spectral decomposition. Neuroimage *55*, 1528–1535.

25. Cohen, M.X. (2022). A tutorial on generalized eigendecomposition for denoising, contrast enhancement, and dimension reduction in multichannel electrophysiology. Neuroimage *247*, 118809.

26. Ramoser, H., Muller-Gerking, J., and Pfurtscheller, G. (2000). Optimal spatial filtering of single trial EEG during imagined hand movement. IEEE Trans. Rehabil. Eng. *8*, 441–446.

27. Wolpaw, J.R., McFarland, D.J., Neat, G.W., and Forneris, C.A. (1991). An EEG-based brain-computer interface for cursor control. Electroencephalogr. Clin. Neurophysiol. *78*, 252–259.

28. Shaw, N.A. (2002). The neurophysiology of concussion. Prog. Neurobiol. *67*, 281–344.

29. Hirano, Y., and Uhlhaas, P.J. (2021). Current findings and perspectives on aberrant neural oscillations in schizophrenia. Psychiatr. Clin. Neurosci. *75*, 358–368.

30. Wu, W., Zhang, Y., Jiang, J., Lucas, M.V., Fonzo, G.A., Rolle, C.E., Cooper, C., Chin-Fatt, C., Krepel, N., Cornelssen, C.A., et al. (2020). An electroencephalographic signature predicts antidepressant response in major depression. Nat. Biotechnol. *38*, 439–447.

31. Mosher, J.C., Leahy, R.M., and Lewis, P.S. (1999). EEG and MEG: Forward solutions for inverse methods. IEEE Trans. Biomed. Eng. *46*, 245–259.

32. Nunez, P.L., and Srinivasan, R. (2006). Electric Fields of the Brain: The Neurophysics of EEG (USA: Oxford University Press).

33. Dähne, S., Meinecke, F.C., Haufe, S., Höhne, J., Tangermann, M., Müller, K.-R., and Nikulin, V.V. (2014). SPoC: A novel framework for relating the amplitude of neuronal oscillations to behaviorally relevant parameters. Neuroimage *86*, 111–122. https://doi.org/10.1016/j.neuroimage.2013.07.079.

34. Sabbagh, D., Ablin, P., Varoquaux, G., Gramfort, A., and Engemann, D.A. (2020). Predictive Regression Modeling with MEG/EEG: From Source Power to Signals and Cognitive States. Neuroimage *222*, 116893. https://doi.org/10.1016/j.neuroimage.2020.116893.

35. Hipp, J.F., Hawellek, D.J., Corbetta, M., Siegel, M., and Engel, A.K. (2012). Large-Scale Cortical Correlation Structure of Spontaneous Oscillatory Activity. Nat. Neurosci. *15*, 884–890. https://doi.org/10.1038/nn.3101.

36. Ranasinghe, K.G., Kudo, K., Hinkley, L., Beagle, A., Lerner, H., Mizuiri, D., Findlay, A., Miller, B.L., Kramer, J.H., Gorno-Tempini, M.L., et al. (2022). Neuronal synchrony abnormalities associated with subclinical epileptiform activity in early-onset Alzheimer's disease. Brain *145*, 744–753.

37. Kudo, K., Ranasinghe, K.G., Morise, H., Syed, F., Sekihara, K., Rankin, K.P., Miller, B.L., Kramer, J.H., Rabinovici, G.D., Vossel, K., et al. (2024). Neurophysiological trajectories in Alzheimer's disease progression. Elife *12*, RP91044.

38. Destrieux, C., Fischl, B., Dale, A., and Halgren, E. (2010). Automatic parcellation of human cortical gyri and sulci using standard anatomical nomenclature. Neuroimage *53*, 1–15.

39. Tait, L., Özkan, A., Szul, M.J., and Zhang, J. (2021). A systematic evaluation of source reconstruction of resting MEG of the human brain with a new high-resolution atlas: Performance, precision, and parcellation. Hum. Brain Mapp. *42*, 4685–4707.

40. Khan, S., Hashmi, J.A., Mamashli, F., Michmizos, K., Kitzbichler, M.G., Bharadwaj, H., Bekhti, Y., Ganesan, S., Garel, K.-L.A., Whitfield-Gabrieli, S., et al. (2018). Maturation trajectories of cortical resting-state networks depend on the mediating frequency band. Neuroimage *174*, 57–68.

41. Koles, Z.J., Lazar, M.S., and Zhou, S.Z. (1990). Spatial patterns underlying population differences in the background EEG. Brain Topogr. *2*, 275–284.

42. Barachant, A., Bonnet, S., Congedo, M., and Jutten, C. (2013). Classification of Covariance Matrices Using a Riemannian-based Kernel for BCI Applications. Neurocomputing *112*, 172–178. https://doi.org/10.1016/j.neucom.2012.12.039.

43. Barachant, A., Bonnet, S., Congedo, M., and Jutten, C. (2010a). Common spatial pattern revisited by Riemannian geometry. In 2010 IEEE International Workshop on Multimedia Signal Processing. IEEE, pp. 472–476.

44. LeCun, Y., Bengio, Y., and Hinton, G. (2015). Deep learning. nature *521*, 436–444.

45. Schmidhuber, J. (2015). Deep learning in neural networks: An overview. Neural Network. *61*, 85–117.

46. Szegedy, C., Liu, W., Jia, Y., Sermanet, P., Reed, S., Anguelov, D., Erhan, D., Vanhoucke, V., and Rabinovich, A. (2015). Going deeper with convolutions. In Proceedings of the IEEE Conference on Computer Vision and Pattern Recognition, pp. 1–9.

47. Schirrmeister, R.T., Springenberg, J.T., Fiederer, L.D.J., Glasstetter, M., Eggensperger, K., Tangermann, M., Hutter, F., Burgard, W., and Ball, T. (2017). Deep Learning with Convolutional Neural Networks for EEG Decoding and Visualization. Hum. Brain Mapp. *38*, 5391–5420. https://doi.org/10.1002/hbm.23730.

48. Lawhern, V.J., Solon, A.J., Waytowich, N.R., Gordon, S.M., Hung, C.P., and Lance, B.J. (2018). Eegnet: a compact convolutional neural network for eeg-based brain–computer interfaces. J. Neural. Eng. *15*, 056013.

49. Kingma, D.P., and Ba, J. (2017). Adam: A Method for Stochastic Optimization. Preprint at arXiv. https://doi.org/10.48550/arXiv.1412.6980.

50. Robbins, H., and Monro, S. (1951). A stochastic approximation method. Ann. Math. Stat. *22*, 400–407.

51. Zhang, C., Kim, Y.-K., and Eskandarian, A. (2021). EEG-inception: An accurate and robust end-to-end neural network for EEG-based motor imagery classification. J. Neural. Eng. *18*, 046014.

52. Şeker, M., and Özerdem, M.S. (2024). Deep insights into MCI diagnosis: A comparative deep learning analysis of EEG time series. J. Neurosci. Methods *403*, 110057. https://doi.org/10.1016/j.jneumeth.2024.110057.

53. Buzsáki, G., and Draguhn, A. (2004). Neuronal oscillations in cortical networks. Science (New York, N.Y.) *304*, 1926–1929. https://doi.org/10.1126/science.1099745.

54. Barmpas, K., Panagakis, Y., Adamos, D.A., Laskaris, N., and Zafeiriou, S. (2023). BrainWave-Scattering Net: A Lightweight Network for EEG-based Motor Imagery Recognition. J. Neural. Eng. *20*, 056014. https://doi.org/10.1088/1741-2552/acf78a.

55. Wilson, D., Schirrmeister, R.T., Gemein, L.A.W., and Ball, T. (2022). Deep Riemannian Networks for EEG Decoding. Preprint at arXiv. https://doi.org/10.48550/arXiv.2212.10426.

56. Carrara, I., Aristimunha, B., Corsi, M.-C., de Camargo, R.Y., Chevallier, S., and Papadopoulo, T. (2024). Geometric Neural Network based on Phase Space for BCI decoding. Preprint at arXiv. https://doi.org/10.48550/arXiv.2403.05645.

57. Liu, W., Guo, C., and Gao, C. (2024). A cross-session motor imagery classification method based on Riemannian geometry and deep domain adaptation. Expert Syst. Appl. *237*, 121612. https://doi.org/10.1016/j.eswa.2023.121612.

58. Zhang, G., and Etemad, A. (2024). Spatio-temporal EEG representation learning on riemannian manifold and euclidean space. IEEE Trans. Emerg. Top. Comput. Intell. *8*, 1469–1483. https://doi.org/10.1109/TETCI.2023.3332549.

59. Lachaux, J.-P., Rodriguez, E., Martinerie, J., and Varela, F.J. (1999). Measuring Phase Synchrony in Brain Signals. Hum. Brain Mapp. *8*, 194–208. https://doi.org/10.1002/(sici)1097-0193(1999)8:4<194::aid-hbm4>3.0.co;2-c.

60. Varela, F., Lachaux, J.P., Rodriguez, E., and Martinerie, J. (2001). The brainweb: Phase synchronization and large-scale integration. Nat. Rev. Neurosci. *2*, 229–239. https://doi.org/10.1038/35067550.

61. Thompson, N.C., Greenewald, K., Lee, K., and Manso, G.F. (2020). The computational limits of deep learning. Preprint at arXiv. https://doi.org/10.48550/arXiv.2007.05558.

62. Thompson, N., Fleming, M., Tang, B.J., Pastwa, A.M., Borge, N., Goehring, B.C., and Das, S. (2024). A model for estimating the economic costs of computer vision systems that use deep learning. Proc. AAAI Conf. Artif. Intell. *38*, 23012–23018. https://doi.org/10.1609/aaai.v38i21.30343.

63. Ledoit, O., and Wolf, M. (2004). A Well-Conditioned Estimator for Large-Dimensional Covariance Matrices. J. Multivariate Anal. *88*, 365–411. https://doi.org/10.1016/s0047-259x(03)00096-4.

64. Huang, Z., and Gool, L.V. (2017). A Riemannian Network for SPD Matrix Learning. Proc. AAAI Conf. Artif. Intell. *31*, 2036–2042. https://doi.org/10.1609/aaai.v31i1.10866.

65. Nadeau, C., Bengio, Y. Inference for the generalization error. Advances in neural information processing systems 12 (1999). Eds: S.A. Solla, T.K. Leen, K. Müller

66. Engemann, D.A., Mellot, A., Höchenberger, R., Banville, H., Sabbagh, D., Gemein, L., Ball, T., and Gramfort, A. (2022). A Reusable Benchmark of Brain-Age Prediction from M/EEG Resting-State Signals. Neuroimage *262*, 119521. https://doi.org/10.1016/j.neuroimage.2022.119521.

67. Bomatter, P., Paillard, J., Garces, P., Hipp, J., and Engemann, D.-A. (2024). Machine learning of brain-specific biomarkers from eeg. EBioMedicine *106*, 105259. https://doi.org/10.1016/j.ebiom.2024.105259.

68. Song, Y., Zheng, Q., Liu, B., and Gao, X. (2023). Eeg conformer: Convolutional transformer for eeg decoding and visualization. IEEE Trans. Neural Syst. Rehabil. Eng. *31*, 710–719.

69. Infantosi, A.F.C., and Miranda de Sá, A.M.F.L. (2006). A coherence-based technique for separating phase-locked from non-phase-locked power spectrum estimates during intermittent stimulation. J. Neurosci. Methods *156*, 267–274.

70. Lakatos, P., Gross, J., and Thut, G. (2019). A new unifying account of the roles of neuronal entrainment. Curr. Biol. *29*, R890–R905.

71. Schroeder, C.E., and Lakatos, P. (2009). Low-frequency neuronal oscillations as instruments of sensory selection. Trends Neurosci. *32*, 9–18.

72. Buzsáki, G. (2006). Rhythms of the Brain (Oxford university press).

73. Tangermann, M., Müller, K.-R., Aertsen, A., Birbaumer, N., Braun, C., Brunner, C., Leeb, R., Mehring, C., Miller, K.J., Müller-Putz, G.R., et al. (2012). Review of the bci competition iv. Front. Neurosci. *6*, 55.

74. Chevallier, S., Carrara, I., Aristimunha, B., Guetschel, P., Sedlar, S., Lopes, B., Velut, S., Khazem, S., and Moreau, T. (2024). The largest eeg-based bci reproducibility study for open science: the moabb benchmark. Preprint at arXiv. https://doi.org/10.48550/arXiv.2404.15319.

75. Sadaghiani, S., Brookes, M.J., and Baillet, S. (2022). Connectomics of human electrophysiology. Neuroimage *247*, 118788.

76. Canolty, R.T., and Knight, R.T. (2010). The functional role of cross-frequency coupling. Trends Cognit. Sci. *14*, 506–515.

77. Jensen, O., and Colgin, L.L. (2007). Cross-frequency coupling between neuronal oscillations. Trends Cognit. Sci. *11*, 267–269.

78. Morlet, J., Arens, G., Fourgeau, E., and Giard, D. (1982). Wave Propagation and Sampling Theory—Part II: Sampling Theory and Complex Waves. Geophysics *47*, 222–236. https://doi.org/10.1190/1.1441329.

79. Corsi, M.-C., Chevallier, S., Fallani, F.D.V., and Yger, F. (2022). Functional connectivity ensemble method to enhance BCI performance (FUCONE). IEEE Trans. Biomed. Eng. *69*, 2826–2838.

80. Wolpert, D.H. (1992). Stacked generalization. Neural Network. *5*, 241–259.

81. Engemann, D.A., Kozynets, O., Sabbagh, D., Lemaître, G., Varoquaux, G., Liem, F., and Gramfort, A. (2020). Combining magnetoencephalography with magnetic resonance imaging enhances learning of surrogate-biomarkers. Elife *9*, e54055.

82. Sabbagh, D., Cartailler, J., Touchard, C., Joachim, J., Mebazaa, A., Vallée, F., Gayat, É., Gramfort, A., and Engemann, D.A. (2023). Repurposing electroencephalogram monitoring of general anaesthesia for building biomarkers of brain ageing: An exploratory study. BJA open *7*, 100145.

83. Das, P., He, M., and Purdon, P.L. (2024). A dynamic generative model can extract interpretable oscillatory components from multichannel neurophysiological recordings. Preprint at bioRxiv, 2023.07.26.550594. https://doi.org/10.1101/2023.07.26.550594.

84. Frohlich, J., Reiter, L.T., Saravanapandian, V., DiStefano, C., Huberty, S., Hyde, C., Chamberlain, S., Bearden, C.E., Golshani, P., Irimia, A., et al. (2019b). Mechanisms underlying the EEG biomarker in Dup15q syndrome. Mol. Autism. *10*, 29. https://doi.org/10.1186/s13229-019-0280-6.

85. Janz, P., Nicolas, M.J., Redondo, R.L., and Valencia, M. (2022). GABAB R activation partially normalizes acute NMDAR hypofunction oscillatory abnormalities but fails to rescue sensory processing deficits. J. Neurochem. *161*, 417–434.

86. Hipp, J.F., Knoflach, F., Comley, R., Ballard, T.M., Honer, M., Trube, G., Gasser, R., Prinssen, E., Wallace, T.L., Rothfuss, A., et al. (2021). Basmisanil, a highly selective GABAA-α5 negative allosteric modulator: Preclinical pharmacology and demonstration of functional target engagement in man. Sci. Rep. *11*, 7700.

87. Hayashi, S., Caron, B.A., Heinsfeld, A.S., Vinci-Booher, S., McPherson, B., Bullock, D.N., Bertò, G., Niso, G., Hanekamp, S., Levitas, D., et al. (2024). Brainlife. io: A decentralized and open-source cloud platform to support neuroscience research. Nat. Methods *21*, 809–813.

88. Renton, A.I., Dao, T.T., Johnstone, T., Civier, O., Sullivan, R.P., White, D.J., Lyons, P., Slade, B.M., Abbott, D.F., Amos, T.J., et al. (2024). Neurodesk: An accessible, flexible and portable data analysis environment for reproducible neuroimaging. Nat. Methods *21*, 804–808.

89. Sabbagh, D., Ablin, P., Varoquaux, G., Gramfort, A., and Engemann, D.A. (2019). Manifold-regression to predict from MEG/EEG brain signals without source modeling. Adv. Neural. Inf. Process. Syst. *32*.

90. Berlinet, A., and Thomas-Agnan, C. (2011). Reproducing Kernel Hilbert Spaces in Probability and Statistics (Springer Science & Business Media).

91. Jayasumana, S., Hartley, R., Salzmann, M., Li, H., and Harandi, M. (2015). Kernel Methods on Riemannian Manifolds with Gaussian RBF Kernels. IEEE Trans. Pattern Anal. Mach. Intell. *37*, 2464–2477. https://doi.org/10.1109/TPAMI.2015.2414422.

92. Wahba, G. (1981). Spline interpolation and smoothing on the sphere. SIAM J. Sci. Stat. Comput. *2*, 5–16.

93. Williams, C., and Rasmussen, C. (1995). Gaussian processes for regression. Adv. Neural Inf. Process. Syst. *8*.

94. Sejdinovic, D., Sriperumbudur, B., Gretton, A., and Fukumizu, K. (2013). Equivalence of distance-based and RKHS-based statistics in hypothesis testing. Ann. Stat. *41*, 2263–2291.

95. Cole, S.R., and Voytek, B. (2017). Brain oscillations and the importance of waveform shape. Trends Cognit. Sci. *21*, 137–149.

96. Jackson, N., Cole, S.R., Voytek, B., and Swann, N.C. (2019). Characteristics of waveform shape in parkinson's disease detected with scalp electroencephalography. eNeuro *6*, 1–11.

97. Bruna, J., and Mallat, S. (2013). Invariant scattering convolution networks. IEEE Trans. Pattern Anal. Mach. Intell. *35*, 1872–1886. https://doi.org/10.1109/tpami.2012.230.

98. Mallat, S. (1999). A Wavelet Tour of Signal Processing (Elsevier).

99. Jing, J., Sun, H., Kim, J.A., Herlopian, A., Karakis, I., Ng, M., Halford, J.J., Maus, D., Chan, F., Dolatshahi, M., et al. (2020). Development of

CellPress
OPEN ACCESS

expert-level automated detection of epileptiform discharges during electroencephalogram interpretation. JAMA Neurol. *77*, 103–108.

100. Vidaurre, D., Quinn, A.J., Baker, A.P., Dupret, D., Tejero-Cantero, A., and Woolrich, M.W. (2016). Spectrally resolved fast transient brain states in electrophysiological data. Neuroimage *126*, 81–95.

101. Bonet, C., Malézieux, B., Rakotomamonjy, A., Drumetz, L., Moreau, T., Kowalski, M., and Courty, N. (2023). In Sliced-Wasserstein on symmetric positive definite matrices for M/EEG signals. International Conference on Machine Learning (PMLR), pp. 2777–2805.

102. Bashivan, P., Rish, I., Yeasin, M., and Codella, N. (2015). Learning representations from EEG with deep recurrent-convolutional neural networks. Preprint at arXiv. https://doi.org/10.48550/arXiv.1511.06448.

103. Yang, C., Westover, M., and Sun, J. (2024). Biot: Biosignal transformer for cross-data learning in the wild. Adv. Neural Inf. Process. Syst. *36*.

104. Louppe, G., Wehenkel, L., Sutera, A., and Geurts, P. (2013). Understanding variable importances in forests of randomized trees. Adv. Neural Inf. Process. Syst. *26*.

105. Barber, R.F., and Candès, E.J. (2015). Controlling the false discovery rate via knockoffs. Ann. Stat. *43*, 2055–2085.

106. Nguyen, B.T., Thirion, B., and Arlot, S. (2022). A conditional randomization test for sparse logistic regression in high-dimension. Adv. Neural Inf. Process. Syst. *35*, 13691–13703.

107. Chamma, A., Engemann, D.A., and Thirion, B. (2024a). Statistically valid variable importance assessment through conditional permutations. Adv. Neural Inf. Process. Syst. *36*.

108. Chamma, A., Thirion, B., and Engemann, D. (2024b). Variable importance in high-dimensional settings requires grouping. Proc. AAAI Conf. Artif. Intell. *38*, 11195–11203.

109. Mi, X., Zou, B., Zou, F., and Hu, J. (2021). Permutation-based identification of important biomarkers for complex diseases via machine learning models. Nat. Commun. *12*, 3008.

110. Noroozi, M., and Favaro, P. (2016). In Unsupervised learning of visual representations by solving jigsaw puzzles. European Conference on Computer Vision (Springer), pp. 69–84.

111. Banville, H., Chehab, O., Hyvärinen, A., Engemann, D.-A., and Gramfort, A. (2021). Uncovering the structure of clinical EEG signals with self-supervised learning. J. Neural. Eng. *18*, 046020.

112. Haraldsen, I.H., Hatlestad-Hall, C., Marra, C., Renvall, H., Maestú, F., Acosta-Hernández, J., Alfonsin, S., Andersson, V., Anand, A., Ayllón, V., et al. (2023). Intelligent digital tools for screening of brain connectivity and dementia risk estimation in people affected by mild cognitive impairment: The AI-Mind clinical study protocol. Front. Neurorob. *17*, 1289406.

113. Dzianok, P., and Kublik, E. (2024). PEARL-Neuro Database: EEG, fMRI, health and lifestyle data of middle-aged people at risk of dementia. Sci. Data *11*, 276.

114. Prado, P., Medel, V., Gonzalez-Gomez, R., Sainz-Ballesteros, A., Vidal, V., Santamaría-García, H., Moguilner, S., Mejia, J., Slachevsky, A., Behrens, M.I., et al. (2023). The BrainLat project, a multimodal neuroimaging dataset of neurodegeneration from underrepresented backgrounds. Sci. Data *10*, 889.

115. Dockès, J., Varoquaux, G., and Poline, J.-B. (2021). Preventing dataset shift from breaking machine-learning biomarkers. Gig. Sanit. *10*, giab055.

116. Mellot, A., Collas, A., Rodrigues, P.L.C., Engemann, D., and Gramfort, A. (2023). Harmonizing and aligning M/EEG datasets with covariance-based techniques to enhance predictive regression modeling. Imaging Neuroscience *1*, 1–23.

117. Guetschel, P., Moreau, T., and Tangermann, M. (2024). S-JEPA: Towards seamless cross-dataset transfer through dynamic spatial attention. Preprint at arXiv 2024. doi:10.48550/arXiv.2403.11772.

118. Roy, Y., Banville, H., Albuquerque, I., Gramfort, A., Falk, T.H., and Faubert, J. (2019). Deep Learning-Based Electroencephalography Analysis: A Systematic Review. J. Neural. Eng. *16*, 051001. https://doi.org/10.1088/1741-2552/ab260c.

119. Gabor, D. (1946). Theory of Communication. Part 1: The Analysis of Information. Journal of the Institution of Electrical Engineers *93*, 429–441. https://doi.org/10.1049/ji-3-2.1946.0074.

120. Hoerl, A.E., and Kennard, R.W. (1970). Ridge regression: Biased estimation for nonorthogonal problems. Technometrics *12*, 55–67.

121. Zeghidour, N., Teboul, O., Quitry, F.d.C., and Tagliasacchi, M. (2021). LEAF: A Learnable Frontend for Audio Classification. Preprint at arXiv. https://doi.org/10.48550/arXiv.2101.08596.

122. Cohen, M. (2014). Analyzing Neural Time Series Data: Theory and Practice (MIT press).

123. Congedo, M., Barachant, A., and Bhatia, R. (2017). Riemannian geometry for EEG-based brain-computer interfaces; a primer and a review. Brain-Computer Interfaces *4*, 155–174. https://doi.org/10.1080/2326263x.2017.1297192.

124. Arsigny, V., Fillard, P., Pennec, X., and Ayache, N. (2007). Geometric Means in a Novel Vector Space Structure on Symmetric Positive-Definite Matrices. SIAM J. Matrix Anal. Appl. *29*, 328–347. https://doi.org/10.1137/050637996.

125. Arsigny, V., Fillard, P., Pennec, X., and Ayache, N. (2006). Log-Euclidean Metrics for Fast and Simple Calculus on Diffusion Tensors. Magn. Reson. Med. *56*, 411–421. https://doi.org/10.1002/mrm.20965.

126. Brooks, D., Schwander, O., Barbaresco, F., Schneider, J.-Y., and Cord, M. (2019). Riemannian batch normalization for spd neural networks. 32. https://doi.org/10.48550/arXiv.1909.02414.

127. Absil, P.-A., Mahony, R., and Sepulchre, R. (2008). Optimization Algorithms on Matrix Manifolds (Princeton University Press).

128. Engemann, D.A., and Gramfort, A. (2015). Automated model selection in covariance estimation and spatial whitening of MEG and EEG signals. Neuroimage *108*, 328–342.

129. Chen, Y., Wiesel, A., Eldar, Y.C., and Hero, A.O. (2010). Shrinkage Algorithms for MMSE Covariance Estimation. IEEE Trans. Signal Process. *58*, 5016–5029. https://doi.org/10.1109/tsp.2010.2053029.

130. Lezcano-Casado, M. (2019). Trivializations for Gradient-Based Optimization on Manifolds. Preprint at arXiv. https://doi.org/10.48550/arXiv.1909.09501.

131. de Cheveigné, A., and Parra, L.C. (2014). Joint decorrelation, a versatile tool for multichannel data analysis. Neuroimage *98*, 487–505.

132. Hyvärinen, A., and Oja, E. (2000). Independent Component Analysis: Algorithms and Applications. Neural Network. *13*, 411–430. https://doi.org/10.1016/s0893-6080(00)00026-5.

133. Chevallier, S., Kalunga, E.K., Barthélemy, Q., and Monacelli, E. (2021). Review of Riemannian Distances and Divergences, Applied to SSVEP-based BCI. Neuroinformatics *19*, 93–106. https://doi.org/10.1007/s12021-020-09473-9.

134. Hendrycks, D., and Gimpel, K. (2016). Gaussian error linear units (gelus). Preprint at arXiv. https://doi.org/10.48550/arXiv.1606.08415.

135. Harati, A., Lopez, S., Obeid, I., Picone, J., Jacobson, M.P., and Tobochnik, S. (2014). The TUH EEG Corpus: A Big Data Resource for Automated EEG Interpretation. In 2014 IEEE Signal Processing in Medicine and Biology Symposium (SPMB) (IEEE), pp. 1–5. https://doi.org/10.1109/spmb.2014.7002953.

136. Gemein, L.A., Schirrmeister, R.T., Boedecker, J., and Ball, T. (2024). Brain Age Revisited: Investigating the State vs. Trait Hypotheses of EEG-derived Brain-Age Dynamics with Deep Learning. Imaging Neuroscience *2*, 1–22.

137. Jas, M., Engemann, D.A., Bekhti, Y., Raimondo, F., and Gramfort, A. (2017). Autoreject: Automated Artifact Rejection for MEG and EEG Data. Neuroimage *159*, 417–429. https://doi.org/10.1016/j.neuroimage.2017.06.030.

138. Ablin, P., Cardoso, J.-F., and Gramfort, A. (2018). Faster ICA under Orthogonal Constraint. 2018 IEEE International Conference on Acoustics, Speech and Signal Processing (ICASSP), pp. 4464–4468.

139. Pion-Tonachini, L., Kreutz-Delgado, K., and Makeig, S. (2019). ICLabel: An automated electroencephalographic independent component classifier, dataset, and website. Neuroimage *198*, 181–197.

140. Li, A., Feitelberg, J., Saini, A.P., Höchenberger, R., and Scheltienne, M. (2022). MNE-ICALabel: Automatically annotating ICA components with ICLabel in Python. J. Open Source Softw. *7*, 4484.

141. Gemein, L.A.W., Schirrmeister, R.T., Chrabąszcz, P., Wilson, D., Boedecker, J., Schulze-Bonhage, A., Hutter, F., and Ball, T. (2020). Machine-Learning-Based Diagnostics of EEG Pathology. Neuroimage *220*, 117021. https://doi.org/10.1016/j.neuroimage.2020.117021.

142. Pedregosa, F., Varoquaux, G., Gramfort, A., Michel, V., Thirion, B., Grisel, O., Blondel, M., Prettenhofer, P., Weiss, R., and Dubourg, V.; Others (2011). Scikit-learn: Machine learning in python. the Journal of machine Learning research *12*, 2825–2830.

143. Golub, G.H., and von Matt, U. (1997). Generalized Cross-Validation for Large-Scale problems. J. Comput. Graph Stat. *6*, 1–34.

144. Altaheri, H., Muhammad, G., and Alsulaiman, M. (2023). Physics-informed attention temporal convolutional network for eeg-based motor imagery classification. IEEE Trans. Ind. Inf. *19*, 2249–2258. https://doi.org/10.1109/TII.2022.3197419.

145. Santamaria-Vazquez, E., Martinez-Cagigal, V., Vaquerizo-Villar, F., and Hornero, R. (2020). Eeg-inception: a novel deep convolutional neural network for assistive erp-based brain-computer interfaces. IEEE Trans. Neural Syst. Rehabil. Eng. *28*, 2773–2782.

146. Carrara, I., and Papadopoulo, T. (2024). Classification of bci-eeg based on the augmented covariance matrix. IEEE (Inst. Electr. Electron. Eng.) Trans. Biomed. Eng. *71*, 2651–2662.

147. Barachant, A., Bonnet, S., Congedo, M., and Jutten, C. (2012). Multiclass brain–computer interface classification by riemannian geometry. IEEE Trans. Biomed. Eng. *59*, 920–928.

148. Barachant, A., Bonnet, S., Congedo, M., and Jutten, C. (2010b). Riemannian geometry applied to bci classification. In International conference on latent variable analysis and signal separation, V. Vigneron, V. Zarzoso, E. Moreau, R. Gribonval, and E. Vincent, eds. (Springer), pp. 629–636.

149. Bouckaert, R.R., and Frank, E. (2004). Evaluating the Replicability of Significance Tests for Comparing Learning Algorithms. Advances in Knowledge Discovery and Data Mining. Lect. Notes Comput. Sci. *3056*, 3–12. https://doi.org/10.1007/978-3-540-24775-3_3.

150. Paillard, J., Engemann, D.A., and Keller, E. (2025). Roche/neuro-green: Publication code. Zenodo. https://doi.org/10.5281/zenodo.14597454.

Patterns, Volume *6*

Supplemental information

GREEN: A lightweight architecture using learnable wavelets and Riemannian geometry for biomarker exploration with EEG signals

Joseph Paillard, Jörg F. Hipp, and Denis A. Engemann

Supplementary Materials - Paillard et al. – GREEN

Supplementary Note S1 - Manifold optimization

Given a SPD input matrix, an unconstrained `BiMap` layer will output a symmetric positive **semi**-definite matrix, which means it can have eigenvalues that are zero. To ensure that the output of the `BiMap` layer is a SPD matrix, with strictly positive eigenvalues, the weights of the `BiMap` can be constrained to be semi-orthogonal. To do so, we used the trivialization for gradient-based optimization on manifolds introduced by Lezcano-Casado[1]. This method consists in parametrizing a manifold in terms of a Euclidean space. In *Theorem 4.3*, the author shows that this approach is theoretically equivalent to using Riemannian gradients[2] which has been used in previous publications using Riemannian geometry and DL for EEG[3,4]. In addition to this theoretical result, we empirically observed that both approaches produce similar results. Given the improvement in computation time and reduced overhead, we opted for the trivialization approach.

Supplementary Results

Table S1: **Statistical comparison of G_2 and G_3 against** `baseline`**.** This table reports the detailed test statistics along with the median score, measured using balanced accuracy for all classification tasks and R^2-score for the age-regression.

**task**	**dataset**	**model**	**median score**	$\mathbf{t_{99}}$	`p-value`
age prediction	TUAB	G_3	0.7211	2.9906	0.0018
		G_2	0.6879	0.8650	0.1946
3-6 pathology classification	TUAB	G_3	0.8379	1.7064	0.0455
		G_2	0.8344	2.4251	0.0086
3-6 EO vs EC	TDBRAIN	G_3	0.8623	2.2305	0.01400
		G_2	0.8223	0.2184	0.4138
3-6 sex prediction	TDBRAIN	G_3	0.8325	-0.0491	0.4804
		G_2	0.8141	1.9805	0.0252
3-6 dementia diagnosis	CAU	G_3	0.6045	3.238332	0.0008
		G_2	0.5893	1.5949	0.0570

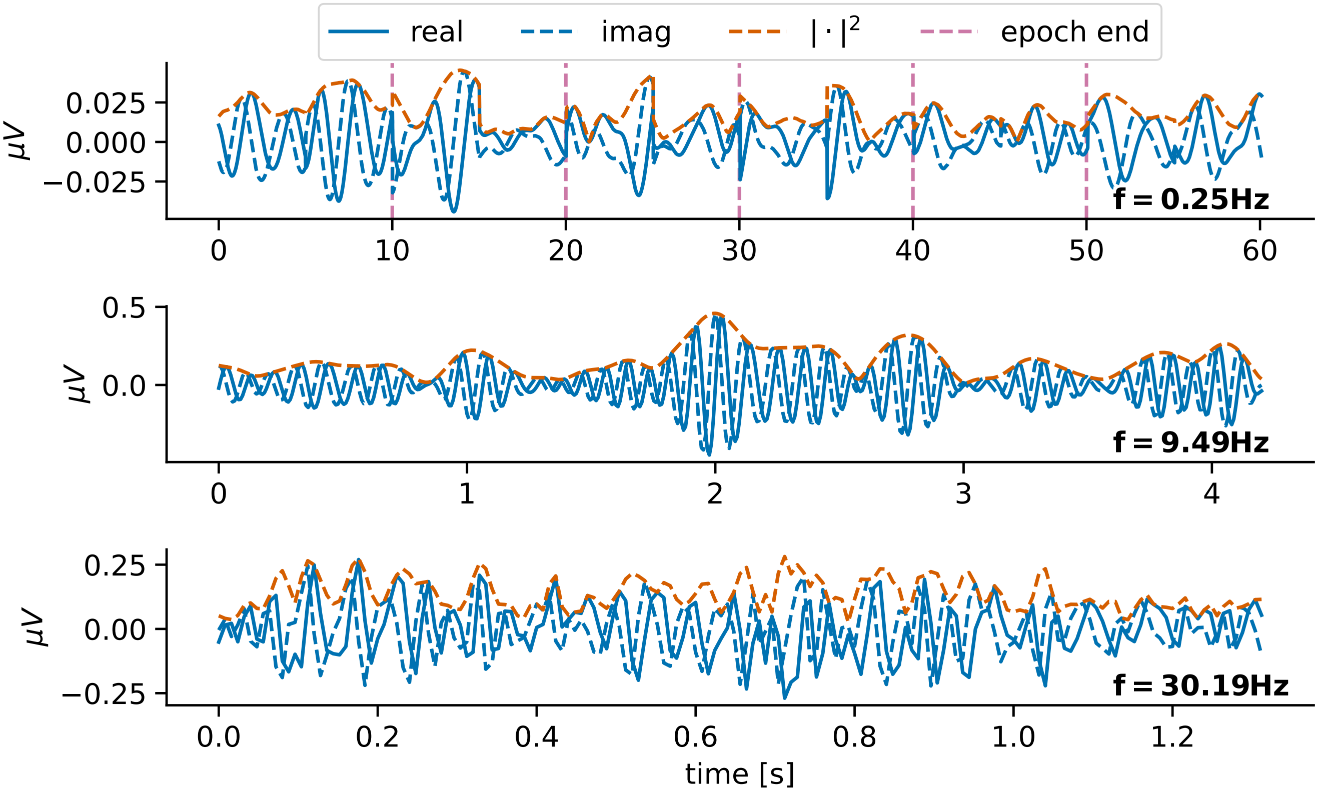

Figure S1: **Wavelet-transformed signal.** Each row represents the output of the convolution by one of the three complex-valued wavelets presented in Figure 5 (main text). The real and the imaginary parts are represented along with the squared modulus, sometimes referred to as power envelope. For the smallest frequency $(0.25Hz)$, multiple epochs have been represented side by side. This plot also reveals the small variations captured by the wavelet, despite the fact that the signal has been high-pass filtered above $1Hz$. This also suggests that task-relevant information is contained around this frequency, therefore pointing a sub-optimal preprocessing choice. The squared modulus variations are slower, evidencing its limited sensitivity to phase variations and small shifts.

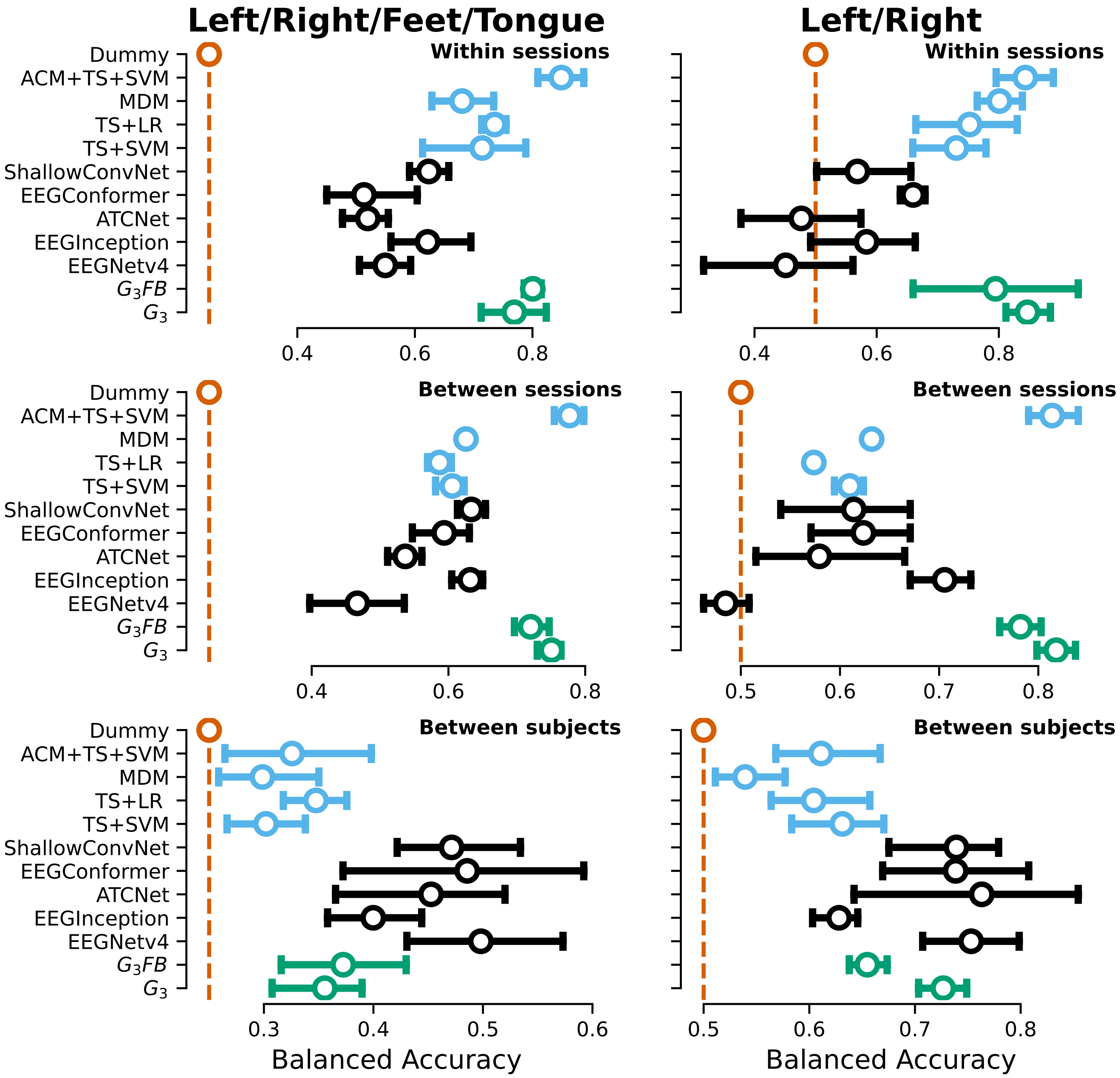

Figure S2: **BCI motor-imagery benchmarks by task and prediction mode (MOABB BNCI2014_001).** Riemannian and Deep architectures compared against GREEN models on motor imagery (left panels) and left/right movement (right panels) for prediction within sessions (first row), between sessions (middle row) and across subjects (bottom row). Chance-level prediction was estimated using a dummy model (red). GREEN models compared favorably for prediction within and between sessions, whereas more complex deep learning architectures showed dominance for motor imagery decoding across subjects. This advantage was less clear for left/right decoding across subjects.

References

1. Lezcano-Casado, M. (2019). Trivializations for Gradient-Based Optimization on Manifolds. arXiv. doi:10.48550/arxiv.1909.09501.

2. Absil, P.-A., Mahony, R., and Sepulchre, R. Optimization Algorithms on Matrix Manifolds. Princeton University Press (2008).

3. Carrara, I., Aristimunha, B., Corsi, M.-C., de Camargo, R. Y., Chevallier, S., and Papadopoulo, T. (2024). Geometric Neural Network based on Phase Space for BCI decoding. arXiv. doi:10.48550/arxiv.2403.05645.

4. Wilson, D., Schirrmeister, R. T., Gemein, L. A. W., and Ball, T. (2022). Deep Riemannian Networks for EEG Decoding. arXiv. doi:10.48550/arxiv.2212.10426.
